# Assessing Biosecurity Risks for the Introduction and Spread of Diseases Among Commercial Sheep Properties in New South Wales, Australia, Using Foot-and-Mouth Disease as a Case Study

**DOI:** 10.3389/fvets.2018.00080

**Published:** 2018-04-17

**Authors:** Jake Fountain, Robert Woodgate, Luzia Rast, Marta Hernández-Jover

**Affiliations:** ^1^Graham Centre for Agricultural Innovation (an alliance between Charles Sturt University and NSW Department of Primary Industries), Wagga Wagga, NSW, Australia; ^2^School of Animal and Veterinary Sciences, Charles Sturt University, Wagga Wagga, NSW, Australia

**Keywords:** risk assessment, biosecurity, sheep, foot-and-mouth disease, Australia

## Abstract

Sheep production systems are a major industry in Australia, with a gross value of roughly $4.66 billion; 87.3% of which is attributable to export markets. Exotic diseases such as foot-and-mouth disease (FMD) are a potential threat to the viability of Australia’s export market. Previous outbreaks of FMD in developed countries, and challenges in the management of onshore biosecurity, signify the importance of on-farm biosecurity in controlling disease transmission. This study aims to investigate the risk of disease introduction and spread among New South Wales (NSW) sheep properties using FMD as a case study and draw recommendation for the industry. Exposure and partial consequence assessments, using scenario trees and Monte Carlo stochastic modeling, were conducted to identify pathways of introduction and spread and calculate the probabilities of these pathways occurring. Input parameters were estimated from the data obtained during qualitative interviews with producers and scientific literature. According to the reported practices of sheep producers and assuming each pathway was carrying the FMD virus, the exposure assessment estimates the median (5–95%) probability of FMD exposure of sheep on a naive property to be 0.619 (0.541–0.698), 0.151 (0.085–0.239), 0.235 (0.153–0.324), and 0.710 (0.619–0.791) for introduction through new stock, wildlife, carriers (humans, dogs, and vehicles), and neighbors, respectively. The spread assessment estimated the median probability of FMD spreading from an infected sheep property to neighboring enterprises to be 0.603 (0.504–0.698). A similar probability was estimated for spread *via* wildlife (0.523; 0.404–0.638); and a lower spread probability was estimated for carriers (0.315; 0.171–0.527), sheep movement (0.285; 0.161–0.462), and dead stock (0.168; 0.070–0.312). The sensitivity analysis revealed that the introduction of an FMD-infected sheep was more influential for exposure *via* new stock than isolation practices. Sharing adjacent boundaries was found to be the most influential factor for exposure and spread between neighboring enterprises, and to a lesser extent, hygiene practices were found to have the most influence on exposure and spread through carriers. To minimize the potential risk of FMD introduction and spread between sheep properties, maintenance of boundary fences, identification of infected animals before introduction to the property, and hygiene and disinfection practices should be improved.

## Introduction

The foot-and-mouth disease (FMD) outbreak experienced by the United Kingdom in 2001 was devastating to the country’s export industry. The world had never witnessed an FMD outbreak in sheep on this scale before, with more than six million animals being slaughtered over the course of the outbreak (1). The direct cost to the private sector after the outbreak was estimated at £5 billion, and export markets to customer countries were ceased for up to 18 months after the United Kingdom was declared free from disease (2). Australia’s sheep production systems rely greatly on the export of live sheep and sheep products, attributing to approximately 87.3% of the value of Australia’s sheep industry at $4.07 billion in 2011 alone (3). Several studies have identified that the estimated cost of a large-scale exotic disease outbreak, such as FMD, to Australia would result in direct loss of $4.3–51.8 billion over 10 years, relating to disease control, domestic product loss, input provider losses (transport and feedstock), and social impacts (increased mental health). The Australian dollar would also fall by 2.5% and remain at this level for the following decade (4, 5).

The involvement of sheep during the 2001 FMD outbreak in the United Kingdom has been well documented in many literary sources (1, 5–7). It was found that sheep were the main transmitter of disease due to the highly variable and often transient nature of FMD clinical signs in this species. Large numbers of sheep were being marketed throughout the country, which resulted in the infection of 48 premises and 15 counties during the first week of the outbreak and before the disease was suspected to be in the country (1). Saleyards in particular have been identified as a risk for the potential of disease amplification and transmission. Sheep movement patterns in Australia are similar to those in the United Kingdom, with 47% of sales through saleyards; sheep may travel at least 200 km from their farm of origin. Furthermore, it is forecasted that saleyards will decrease in number over the next decade, resulting in the transportation of sheep over a greater distance (3). This increased risk for disease transmission in the sheep industry illustrates the importance of adequate on-farm biosecurity and surveillance to limit the potential spread of exotic and endemic diseases.

Palmer (8) identified that the remoteness of Australian farms makes it impossible for members of the agricultural industry to regularly assess the health of every animal on farm. As detection of many diseases, such as FMD, relies on individual inspection of animals, there is an increased necessity for individual producers to implement appropriate on-farm biosecurity practices and to be aware of their responsibilities in relation to the recognition and reporting of disease. On-farm biosecurity practices among livestock producers in Australia have been previously investigated. One of the first studies on biosecurity among rural livestock producers was conducted by Barclay (6). The study found that only half of their respondents had implemented biosecurity measures on their property and that isolating new stock for a short period of time to check for disease was the most common practice. Barclay (6) also found that factors limiting the adoption of biosecurity practices include lack of money, time, information, and property size. While the findings of this study were extensive, biosecurity practices of sheep producers in specific were not represented. Palmer (8) investigated the motivations behind sheep producer’s decisions in relation to biosecurity and reporting in Western Australia. The study found that producer decision-making is based on risk assessment, level of control, degree of self-efficacy in detecting disease and their level of trust in others. The study also discovered that producers did not feel susceptible to disease outbreaks due to a high degree of perceived control and were therefore unlikely to see biosecurity practices as important or applicable. Lack of trust in the government was also found to contribute to producers relying on their own abilities. Local communities were identified as a more trusted source of information. A more recent study undertaken by Taylor et al. (9) assessed the biosecurity practices of sheep producers in relation to the uptake of the National Sheep Health Statement (SHS). The study identified that sheep quarantine and regular monitoring of sheep are not widely implemented, as well as hygiene/cleanliness practices. While this study investigated sheep biosecurity practices in great detail, the relevance of these practices in relation to disease introduction and spread was not investigated.

Recently, other studies have also assessed biosecurity attitudes and practices of cattle producers (10) and smallholder livestock producers (11). Findings indicate that the lack of coordination and collaboration across agencies and organizations in relation to emergency animal disease (EAD) activities (12) has resulted in confusion from a cattle producer perspective. Results of this study indicate that although cattle producers have a high awareness of disease risks to their properties, biosecurity planning and EAD management are given low priority unless there is commercial incentive to address them (10). The biosecurity practices and awareness of EAD among Australian smallholders are poor compared to the commercial sector and the previous study indicated that a better understanding of the communication networks of these producers was needed to improve biosecurity extension delivery (10).

The aim of this study is to investigate the risk of disease introduction and spread among commercial sheep producers in New South Wales (NSW), Australia, using FMD as a case study. To achieve this, the probability of FMD introduction and spread between commercial sheep properties were quantitatively estimated. The study also aimed to identify the most influential biosecurity practices on the risk of disease introduction and spread and provide practical recommendations for the industry.

## Materials and Methods

### Exposure and Spread Assessment Models

An exposure and partial consequence assessment were used in this study, following the OIE methodology for risk analysis (13). The exposure assessment evaluated the potential pathways by which a commercial flock of sheep can be exposed to FMD, and estimated the probability of each of these pathways occurring, assuming that the virus is in the country and present at the origin of each of these pathways and has not yet been identified.

The partial consequence assessment investigated pathways of spread from a mob in which FMD has been introduced and the infection established to other susceptible livestock enterprises. The consequence assessment also estimated the probability of the spread pathways occurring.

The exposure and consequence pathways were represented using scenario trees (14) and developed using Excel (Microsoft 2010). Following the scenario tree methodology for risk analysis, the probability rules were applied when calculating pathway probabilities. Within each scenario of exposure or spread, the node probabilities in each limb of the scenario are conditional on all previous node probabilities for that limb (14), and as such, the multiplication probability rule apply when calculating the probability of positive outcome for each limb (e.g., probability of exposure or spread). For some of the pathway scenarios, several tree limbs lead to a positive outcome (e.g., exposure or spread). The overall probability of exposure and spread for each pathway considered in this assessment was obtained by adding all limb outcome probabilities leading to a positive outcome, as these limbs are considered independent and can all occur at the same time (14). The overall pathway probability is interpreted as the probability of the pathway exposure by occurrence of one or more of the limb events. Probability of exposure and spread were estimated using Monte Carlo stochastic simulation modeling with @Risk 6.0 (Palisade Corporation, USA). Each simulation consisted of 50,000 iterations using the Latin hypercube methods for sampling with a fixed random seed of one.

#### Data Sources

Input values for this model were collected from two main studies investigating the biosecurity practices of Australian sheep producers: a previously published study among 870 sheep producers with more than 100 head of sheep (9) and a qualitative study conducted for the purposes of this assessment and described below. Other scientific literature was used as required.

##### Qualitative Interviews of Sheep Producers in NSW

To complement quantitative biosecurity information gathered in the study by Taylor et al. (9), a qualitative study that was conducted with 12 commercial sheep producers in NSW, Australia, in August 2015. Producers were recruited from those attending a University/Government research sheep forum and through an advertisement published in a government (Local Land Services) newsletter. Face-to-face qualitative interviews, using a semi-structured questionnaire with 48 questions, were used to ask sheep producers about husbandry, biosecurity and animal health practices, communication networks, and EAD knowledge. Criteria for the selection of these participants were limited to production systems with more than 500 head of sheep in total within 270 km radius of Wagga Wagga, NSW. The interview was recorded and subsequently transcribed into Microsoft Excel (PC/Windows XP, 2007). Each participant received an AU$100 gift voucher on completion of the interview. Descriptive statistics were conducted for quantitative data collected during the interviews, and qualitative data were analyzed using content analysis to identify themes and connections between producer responses. These data were used to parameterize input values in the current study’s models. The questionnaire and interview process were approved by the Human Ethics Low Risk Committee of Charles Sturt University, Australia (Approval 400/2015/28). The questionnaire is available from the corresponding author on request.

#### Exposure Assessment

**Figure 1:**
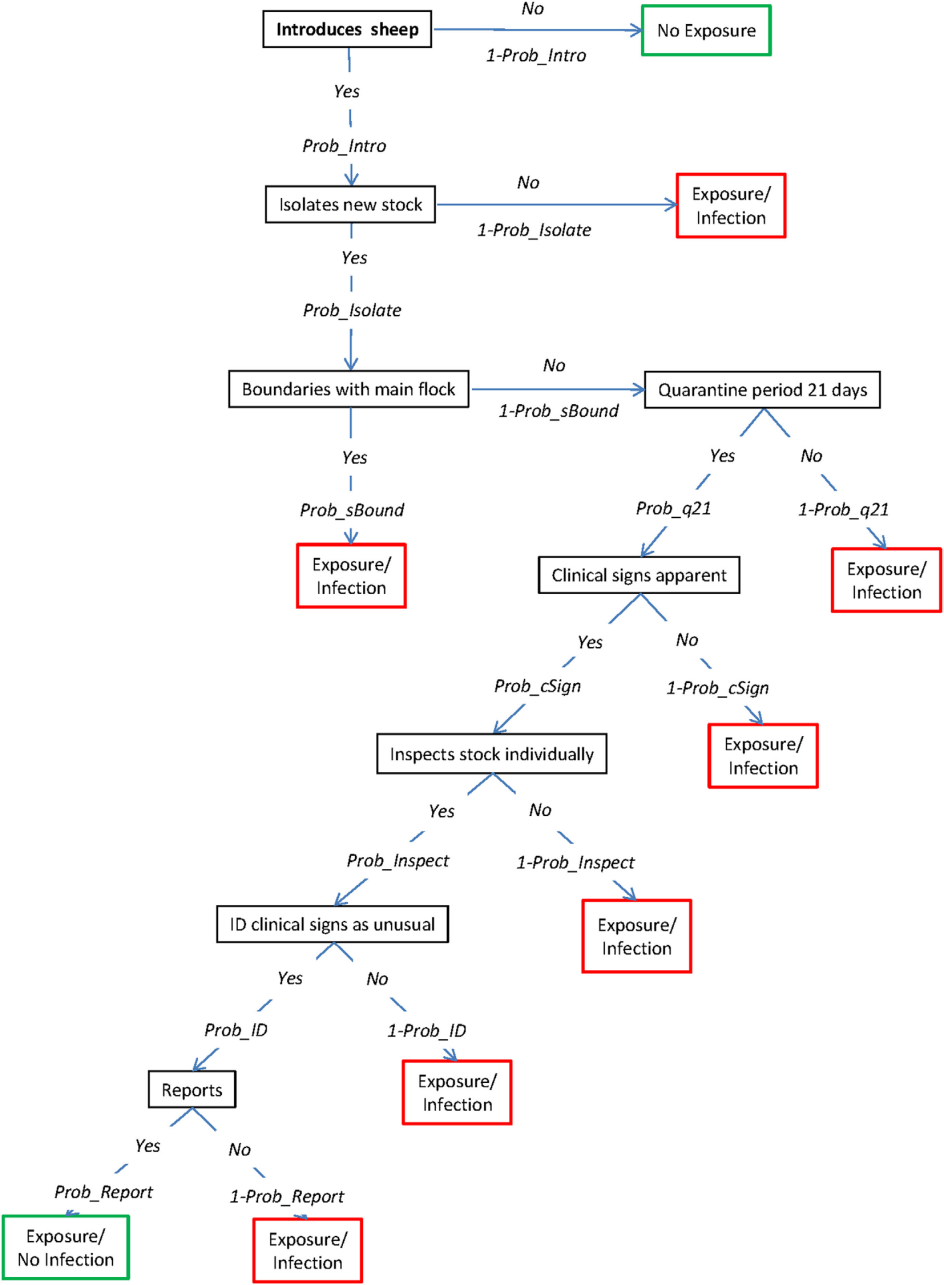
Scenario tree representing the pathways of exposure of sheep to foot-and-mouth disease (FMD) through the introduction of infected sheep. (Prob_Intro, probability that a producer will introduce a FMD-infected sheep; Prob_Isolate, probability that a producer will isolate introduced stock; Prob_sBound, probability of an isolated animal sharing boundaries with flock; Prob_q21, probability that quarantine will last for 21 days; Prob_cSign, probability of sheep developing clinical signs; Prob_Inspect, probability that a producer will individually inspect new sheep; Prob_ID, probability that a producer will identify FMD-specific lesions as unusual; Prob_Report, probability that producer will contact veterinarian or government agency).

**Figure 2:**
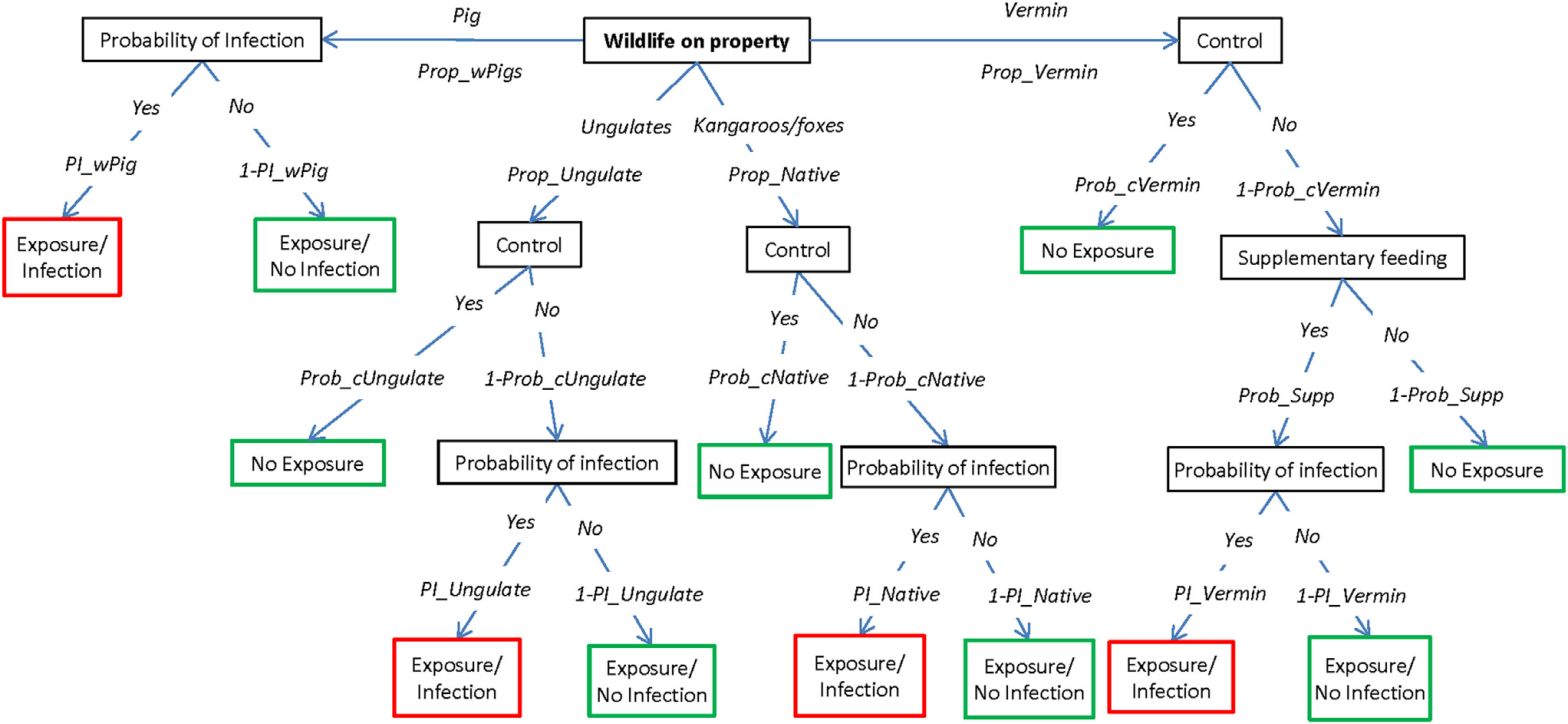
Scenario tree representing the pathways of exposure of sheep to foot-and-mouth disease through contact with wildlife. (Prop_wPigs, proportion of wild pigs on the property; Prop_Ungulate, proportion of deer and goats on the property; Prop_Native, proportion of kangaroos and foxes on the property; Prop_Vermin, proportion of rodents on the property; Prob_cUngulate, probability that producers will control wild deer and goats; Prob_cNative, probability that producers will control kangaroos and foxes; Prob_cVermin, probability that producers will control rodents; Prob_Supp, probability that producers supplementary feed; PI_wPig, probability of infection from wild pigs; PI_Ungulate, probability of infection from deer and goats; PI_Native, probability of infection from kangaroos and foxes; PI_Vermin, probability of infection from rodents).

**Figure 3:**
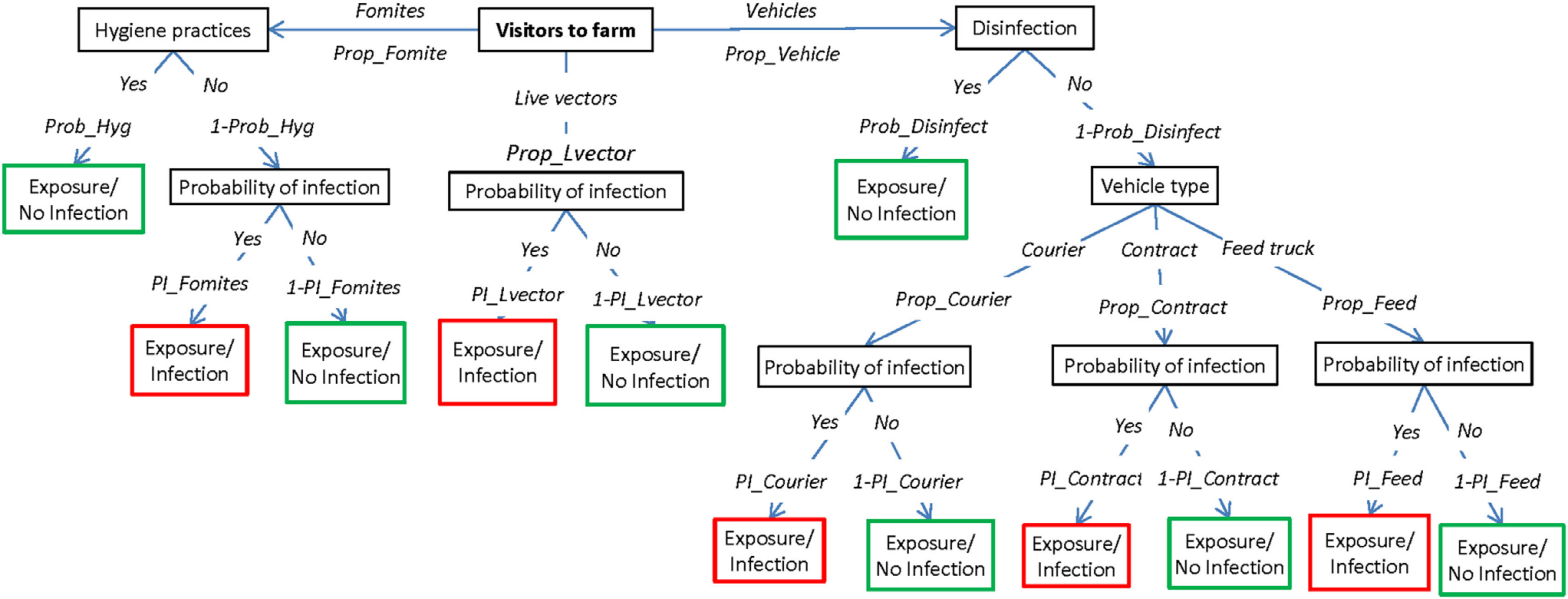
Scenario tree representing the pathways of exposure of sheep to foot-and-mouth disease through potential carriers (humans, dogs, and vehicles). (Prop_Fomite, proportion of fomites entering a property; Prop_Lvector, proportion of humans and dogs entering the property; Prop_Vehicle, proportion of vehicles entering a property; Prob_Hyg, probability that external personnel will take hygiene precautions between properties; Prob_Disinfect, probability that vehicles will be disinfected before entering a property; Prop_Courier, proportion of vehicles that are stock movement vehicles; Prop_Contract, proportion of vehicles that are contractor vehicles; Prop_Feed, proportion of vehicles that are feed trucks; PI_Fomites, probability of infection from fomites; PI_Lvector, probability from infection of humans and dogs; PI_Courier, probability of infection from stock movement vehicles; PI_Contract, probability of infection from contractor vehicles; PI_Feed, probability of infection from feed trucks).

**Figure 4:**
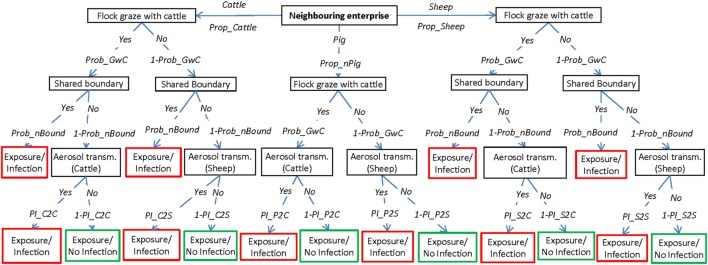
Scenario tree representing the pathways of exposure of sheep to foot-and-mouth disease through livestock on neighboring enterprises. (Prop_Cattle, proportion of neighbors with cattle; Prop_nPigs, proportion of neighbors with domestic pigs; Prop_Sheep, proportion of neighbors with only sheep; Prob_GwC, probability that sheep graze with cattle; Prob_nBound, probability that a sheep flock shares a boundary with an adjacent livestock enterprise; PI_C2C, probability of aerosol transmission from cattle to cattle; PI_C2S, probability of aerosol transmission from cattle to sheep; PI_P2C, probability of aerosol transmission from pigs to cattle; PI_ P2S, probability of aerosol transmission from pigs to sheep; PI_S2C, probability of aerosol transmission from sheep to cattle; PI_ S2S, probability of aerosol transmission from sheep to sheep).

The exposure assessment describes the potential pathways of introduction of FMD to a susceptible sheep property once the disease has been introduced into Australia but has not yet been detected and investigates the relative importance of these pathways for the introduction of the virus. This assessment considers four main pathways by which the FMD virus could be introduced into an individual sheep property, which are represented by four scenario trees: (1) introduction of an FMD-infected animal; (2) contact with wildlife harboring the virus; (3) contact with visitors (humans, canines, and vehicles) carrying the virus; and (4) exposure from an infected neighboring livestock property (Figures 1–4, respectively). A description of the nodes and input parameters for each of the scenario trees are provided in Tables 1–4 and in the online Supplementary Material.

**Table 1:** Nodes, parameter estimates, and input values used for the exposure assessment evaluating the exposure of sheep to foot-and-mouth disease (FMD) through the introduction of infected sheep among commercial sheep properties in New South Wales, Australia.

Node	Branch of node	Parameter estimates	Input value	Data sources
1. Introduces sheep	Yes No	Probability that a producer will introduce an animal in a given year (Prob_Intro)	1- [Pert (0.25, 0.37, 0.5)] Minimum: 882 total sheep producers, 217 producers did not introduce sheep in past 2 years Maximum: 70% of smallholders did not introduce sheep; commercial producers estimated to purchase sheep more often (50%) Most likely: midpoint between maximum and minimum (0.37)	Qualitative study among 12 commercial sheep producers in New South Wales (Australia) (9, 10)
2. Isolates new stock	Yes No	Probability that a producer will isolate stock when it is brought onto the property (Prob_Isolate)	Beta (*s* + 1, *n* − *s* + 1) 881 sheep producers (*n*); 583 isolate new stock on arrival (*s*)	Qualitative study among 12 commercial sheep producers in New South Wales (Australia) (9)
3. Shares boundary with main flock	Yes No	Probability of an isolated animal being held in an enclosure adjacent to a susceptible animal (Prob_sBound)	Beta (*s* + 1, *n* − *s* + 1) 8 sheep producers that quarantine (*n*); 6 producers place quarantined stock adjacent to main flock (*s*)	Qualitative study among 12 commercial sheep producers in New South Wales (Australia)
4. Quarantine period of 21 days	Yes No	Probability that a producer will quarantine introduced animals for a long enough period to allow for detection of FMD clinical signs (Prob_q21)	Pert (0.17, 0.52, 0.88) Minimum: 76 small holders isolated sheep; 13 isolated for ≥21 days. Maximum: 8 sheep producers isolate; 7 isolate for ≥21 days Most likely: midpoint between minimum and maximum.	Qualitative study among 12 commercial sheep producers in New South Wales (Australia) (11)
5. Clinical signs of FMD apparent in sheep	Yes No	Probability of FMD clinical signs developing in infected sheep (Prob_cSign)	Prob_cSign = Moderate (uniform (0.3, 0.7))	(15–17)
6. Inspects new stock individually	Yes No	Probability that a sheep producer will individually inspect introduced animals placed in quarantine (Prob_Inspect)	Uniform (0.42, 0.90) Minimum: 12 sheep producers in total, 5 producers inspect new stock individually Maximum: 870 sheep producers in total, 780 producers inspect new stock	Qualitative study among 12 commercial sheep producers in New South Wales (Australia) (9)
7. Identifies clinical signs as unusual	Yes No	Probability that a sheep producer will identify FMD-specific clinical signs as unusual (Prob_ID)	Prob_ID = Moderate (uniform (0.3, 07))	Qualitative study among 12 commercial sheep producers in New South Wales (Australia) (17)
8. Reports to private veterinarian of government agency	Yes No	Probability that a sheep producer will contact a private veterinarian or government official once unusual signs have been identified (Prob_Report)	Pert (0.5, 0.7, 0.85) Minimum: 12 sheep producers in total, 6 producers contact government for unusual clinical signs. Most likely: 882 sheep producers in total, 619 contact veterinarians for unusual clinical signs Maximum: Average number of producers contacting veterinarians for unusual clinical signs including sheep producers (0.7), beef producers (0.98), and producers from qualitative interviews (0.75)	Qualitative study among 12 commercial sheep producers in New South Wales (Australia) (9, 10)

**Table 2:** Nodes, parameter estimates, and input values used for the exposure assessment evaluating the exposure of sheep to foot-and-mouth disease (FMD) through contact with wildlife among commercial sheep properties in New South Wales, Australia.

Node	Branch of node	Parameter estimates	Input value	Data sources
1. Wildlife found on property	Pigs Ungulates Kangaroos/foxes Vermin	Proportion of wildlife, which commercial NSW sheep producers are exposed to including pigs (Prop_wPigs), ungulates (Prop_Ungulate), kangaroos and foxes (Prop_Native) and rodents (Prop_Vermin)	Beta (*s* + 1, *n* − *s* + 1) 30 potential contacts with wildlife (*n*): 3 reported contact with pigs (*s*), 3 reported contact with ungulates (*s*), 12 reported contact with kangaroos and foxes (*s*), and 12 reported contact with rodents (*s*)	Qualitative study among 12 commercial sheep producers in New South Wales (Australia)
2. Control of wildlife	Yes No	Probability that there is an appropriate control method for wildlife including ungulates (Prob_cUngulate), kangaroos and foxes (Prob_cNative), and rodents (Prob_cVermin)	Beta (*s* + 1, *n* − *s* + 1) Ungulates: 3 reported contact (*n*), 0 reported control (*s*); Kangaroos/Foxes: 12 reported contact (*n*), 5 reported control (*s*); Vermin: 12 reported contact (*n*), 5 reported control (*s*)	As node 1
3. Supplementary feed provided to sheep	Yes No	Probability that supplementary feed will be provided to commercial sheep (Prob_Supp)	Beta (*s* + 1, *n* − *s* + 1) 12 producers (*n*), 6 supplementary feed (s)	As node 1
4. Probability of infection	Yes No	Probability of infection of FMD from ungulates (PI_Ungulate), kangaroos and foxes (PI_Native), rodents (PI_Vermin), and pigs (PI_wPigs) to sheep after exposure has occurred	PI_Ungulate = Moderate (uniform (0.3, 0.7)) PI_Native = Very low (uniform (0.001, 0.05)) PI_Vermin = Very low (uniform (0.001, 0.05)) PI_wPigs = High (uniform (0.7, 1))	(5–7, 16, 17)

**Table 3:** Nodes, parameter estimates, and input values used for the exposure assessment evaluating the exposure of sheep to foot-and-mouth disease (FMD) through potential carriers among commercial sheep properties in New South Wales, Australia.

Node	Branch of node	Parameter estimates	Input value	Data sources
1. Visitors to the property	Live vectors Fomites Vehicles	Proportion of pathways in which the FMD virus could be introduced to a sheep property including humans and dogs (Prop_Lvector), fomites (Prop_Fomite), and vehicles (Prop_Vehicle)	Live vectors: Pert (4, 20, 45) (l); fomites: Pert (1, 17, 43) (f); vehicles: Pert (4, 8, 25) (v). Prop_Lvector = *l*/(*l* + *f* + *v*) Prop_Fomite = *f*/(*l* + *f* + *v*) Prop_Vehicle = *v*/(*l* + *f* + *v*)	Qualitative study among 12 commercial sheep producers in New South Wales (Australia)
2. Hygiene practices	Yes No	Probability that external personnel will take hygiene precautions between properties (Prob_Hyg)	Uniform (0.16, 0.53) 12 producers, 2 producers request clean equipment 870 producers; 465 ensure equipment is clean	Qualitative study among 12 commercial sheep producers in New South Wales (Australia) (9)
3. Disinfection of vehicles	Yes No	Probability of a contractor, feed truck, and stock movement vehicle being disinfected before coming into contact with sheep and sheep areas on the property (Prob_Disinfect)	Uniform (0.10, 0.33) 215 contractor vehicles, 22 clean their vehicles 12 producers, 4 producers ensure vehicles are clean	Qualitative study among 12 commercial sheep producers in New South Wales (Australia) (18)
4. Type of vehicle	Livestock vehicle Contractors Feed truck	Proportion of vehicle types that may enter a sheep property including stock movement vehicles (Prop_Courier), personnel (Prop_Contract), and feed trucks (Prop_Feed)	Beta (*s* + 1, *n* − *s* + 1) 177 vehicles (*n*), 54 livestock transport vehicles (*s*); 107 personnel vehicles (*s*); 16 supplementary feed trucks (*s*)	As node 1
5. Probability of infection	Yes No	Probability of sheep being exposed and infected with FMD from fomites (PI_Fomites), humans and dogs (PI_Lvector), livestock movement vehicles (PI_Courier), personnel (PI_Contract), and supplementary feed vehicles (PI_Feed)	PI_Fomites = Moderate (uniform (0.3, 0.7)) PI_Lvector = Low (uniform (0.05, 0.3)) PI_Courier = Moderate (uniform (0.3, 0.7)) PI_Contract = Low (uniform (0.05, 0.3)) PI_Feed = Very Low (uniform (0.001, 0.05))	(1, 2, 5, 7, 17, 18)

**Table 4:** Nodes, parameter estimates, and input values used for the exposure assessment evaluating the exposure of sheep to foot-and-mouth disease (FMD) through livestock on neighboring enterprises among commercial sheep properties in New South Wales, Australia.

Node	Branch of node	Parameter estimates	Input value	Data sources
1. Neighboring enterprises	Pig Cattle Sheep	Proportion of livestock enterprises surrounding sheep properties including pigs (Prop_nPig), cattle (Prop_Cattle), and sheep (Prop_Sheep) enterprises	Beta (*s* + 1, *n* − *s* + 1) 49 neighboring enterprises in total (*n*); 6 pig enterprises (*s*); 32 sheep enterprises (*s*); 11 cattle enterprises (*s*)	Qualitative study among 12 commercial sheep producers in New South Wales (Australia)
2. Sheep flock graze with cattle	Yes No	Probability that sheep flocks are held on the same pastures as cattle (Prob_GwC)	Beta (*s* + 1, *n* − *s* + 1) 187 producers in total (*n*); 106 run cattle and sheep together (*s*)	Qualitative study among 12 commercial sheep producers in New South Wales (Australia) (10)
3. Shared boundary with neighboring stock	Yes No	Probability that a sheep flock will share a boundary with an adjacent livestock enterprise (Prob_nBound)	Beta (*s* + 1, *n* − *s* + 1) 50 boundaries (*n*); 34 boundaries adjacent with neighboring stock (*s*)	Qualitative study among 12 commercial sheep producers in New South Wales (Australia)
4. Exposure via aerosol	Yes No	Probability that livestock will be exposed to and infected with FMD *via* aerosol from cattle to cattle (PI_C2C), pigs to cattle (PI_P2C), pigs to sheep (PI_P2S), sheep to cattle (PI_S2C), sheep to sheep (PI_S2S), and cattle to sheep (PI_C2S)	PI_C2C = Low (uniform (0.05, 0.3)) PI_P2C = High (uniform (0.7, 1)) PI_P2S = Moderate (uniform (0.3, 0.7)) PI_S2C = Low (uniform (0.05, 0.3)) PI_S2S = Very low (uniform (0.001, 0.05)) PI_C2S = Very low (uniform (0.001, 0.05))	(17, 19)

#### Probability of Spread

**Figure 5:**
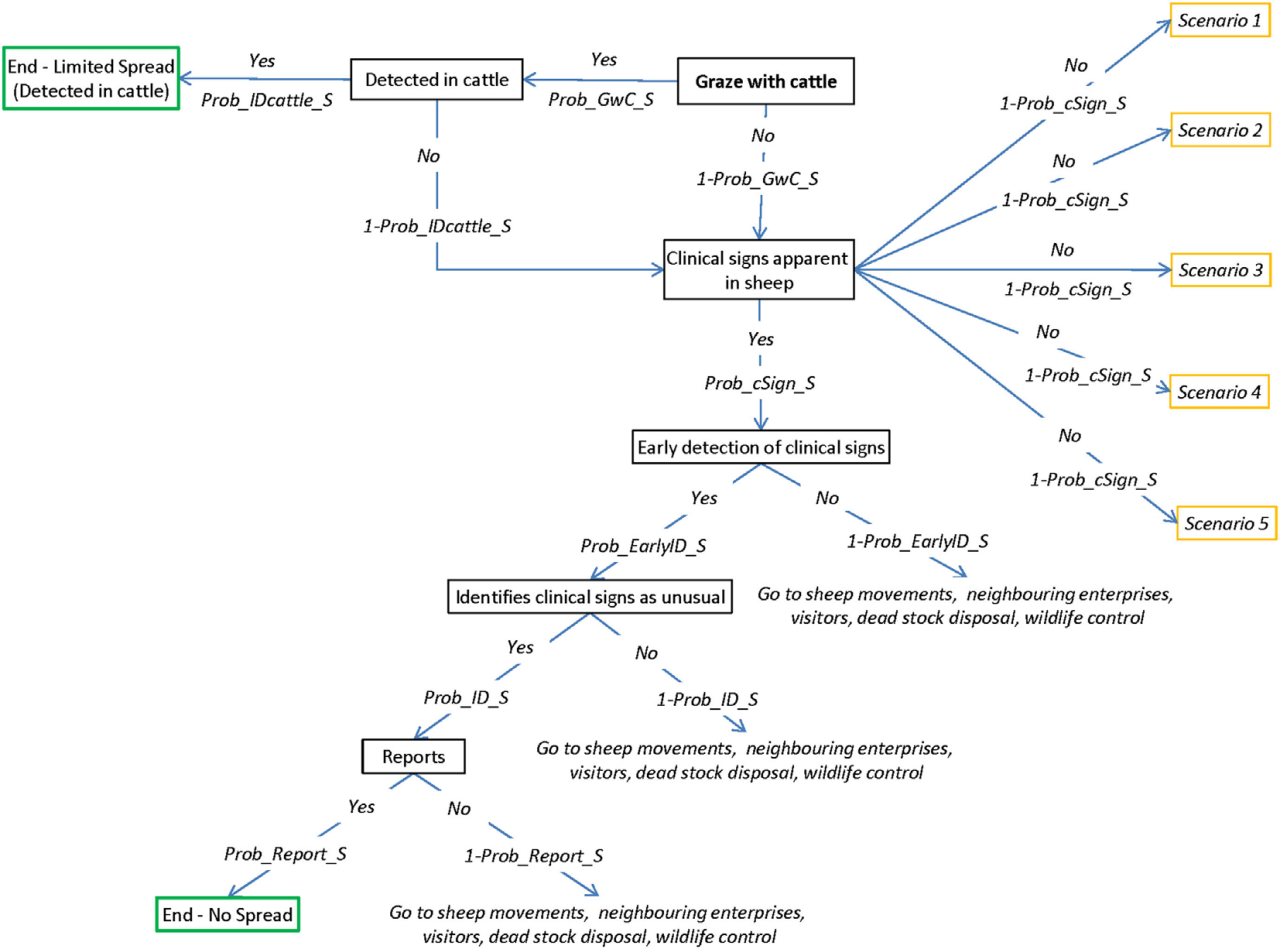
Scenario tree representing the overall spread of foot-and-mouth disease (FMD) from an infected sheep property to other susceptible livestock enterprises. (Prob_GwC_S, probability that sheep graze with cattle; Prob_IDcattle_S, probability that FMD will be detected in cattle; Prob_cSigns_S, probability of sheep developing clinical signs; Prob_EarlyID_S, probability that FMD will be detected in sheep early enough to limit disease spread; Prob_ID_S, probability that a producer will identify FMD-specific lesions as unusual; Prob_Report_S, probability that producer will contact veterinarian or government agency).

A partial consequence assessment was conducted, which described potential pathways of FMD spread from an infected sheep property to other susceptible livestock in other enterprises. Impacts of this spread were not investigated in this consequence assessment. Five main spread pathways were considered, including (1) spread *via* live sheep movements; (2) spread by visitors exiting the property; (3) spread to neighboring enterprises; (4) spread *via* infected carcass material; (5) spread *via* various wildlife species; and (6) no spread or limited spread. Figure 5 represents the overall spread scenario tree. Node descriptions and input parameters can be found in Table 5 and the online Supplementary Material. Figures representing each of the pathways of spread are provided in the online Supplementary Material.

**Table 5:** Nodes, parameters estimates and input values used for the consequence assessment evaluating the probability of FMD spread from an infected sheep property to other susceptible livestock enterprises.

Node	Branch of node	Parameter estimates	Input value		Data sources
1. Sheep flock graze with cattle	Yes No	Probability that sheep flocks are held on the same pastures as cattle (Prob_GwC_S)	Beta (*s* + 1, *n* − *s* + 1) 187 producers in total (*n*); 106 run cattle and sheep together (*s*)		Qualitative study among 12 commercial sheep producers in New South Wales (Australia) (10)

2. FMD detected in cattle	Yes No	Probability that FMD will be detected in grazing cattle infected with the virus before spread occurs (Prob_IDcattle_S)	Uniform (minimum, maximum) Minimum: Overall probability of daily inspection (DI)/y = Σ (Proportion of producers inspecting sheep daily × Proportion of weeks/y of DI) Beta (*s* + 1, *n* − *s* + 1) 1/12 producer × 18/52 weeks DI 6/12 producers × 6/52 weeks DI 5/12 producers × 0/52 weeks DI Maximum: Beta (*s* + 1, *n* − *s* + 1) 181 beef producers (*n*), 50 inspect cattle daily (*s*)		Qualitative study among 12 commercial sheep producers in New South Wales (Australia) (10)

3. Clinical signs of FMD apparent in sheep	Yes No	Probability of FMD clinical signs developing in infected sheep (Prob_cSign_S)	Prob_cSign_S = Moderate (Uniform (0.3, 0.7))		(15–17)

4. Early detection of clinical signs	Yes No	Probability that FMD will be detected in sheep early enough to prevent spread of FMD (Prob_EarlyID_S)	Overall probability of DI/y = Σ (Proportion of producers inspecting sheep daily × Proportion of weeks/y of DI) Beta (*s* + 1, *n* − *s* + 1) 1/12 producer × 18/52 weeks DI 6/12 producers × 6/52 weeks DI 5/12 producers × 0/52 weeks DI		Qualitative study among 12 commercial sheep producers in New South Wales (Australia)

5. Identifies clinical signs as unusual	Yes No	Probability that a sheep producer will identify FMD-specific clinical signs as unusual (Prob_ID_S)	Prob_ID_S = Moderate (uniform (0.3,07))		Qualitative study among 12 commercial sheep producers in New South Wales (Australia) (17)

6. Reports to private veterinarian or government agency	Yes No	Probability that a sheep producer will contact a private veterinarian or government official once unusual signs have been identified (Prob_Report_S)	Pert (0.5, 0.7, 0.85) Minimum: 12 sheep producers in total, 6 contact government for unusual clinical signs Most likely: 882 sheep producers in total, 619 contact veterinarians for unusual clinical signs Maximum: Average number of producers contacting veterinarians for unusual clinical signs including sheep (0.7), beef (0.98), and qualitative interview producers (0.75)		Qualitative study among 12 commercial sheep producers in New South Wales (Australia) (9, 10)

7. Spread of FMD	Yes No	Probability of spread through sheep movements (Prob_Mov_S), or visitors (Prob_Visitor_S)	Prob_Mov_S = Σ (Proportion of producers moving sheep/frequency × Qualitative estimate of risk of spread) Beta (*s* + 1, *n* − *s* + 1): Proportion of producers moving sheep/frequencyUniform (min, max): Qualitative estimate of risk of spread		Qualitative study among 12 commercial sheep producers in New South Wales (Australia) (9, 17)
			**Sheep movements/y (*n* producers)**	**Probability of FMD spread**	
			1 (1)	Extremely low (Uniform (0.00001, 0.001))	
			2–4 (3)	Very low (Uniform (0.001, 0.05))	
			5–10 (4)	Low (Uniform (0.05, 0.3))	
			11–20 (4)	Moderate (Uniform (0.3, 0.7))	
			>20 (1)	High (Uniform (0.7, 1))	
			*Prob_Visitor_S* = Σ (Proportion of producers with visitors/frequency × Qualitative estimate of risk of spread)		
			Beta (*s* + 1, *n* − *s* + 1): Proportion of producers moving sheep/frequency Uniform (min, max): Qualitative estimate of risk of spread		
			**Visitor movements/y (*n* producers)**	**Probability of FMD spread**	
			2–4 (2)	Very low (Uniform (0.001, 0.05))	
			5–10 (5)	Low (Uniform (0.05, 0.3))	
			11–20 (2)	Moderate (Uniform (0.3, 0.7))	
			>20 (2)	High (Uniform (0.7, 1))	

8. Destination of sale stock	Abattoir Saleyard Farm	Proportion of a sheep moved off a property to abattoirs (Prop_Abs_S), saleyards (Prop_Saleyard_S), and direct to farm (Prop_Farm_S)	Beta (*s* + 1, *n* − *s* + 1) 114 sheep movements (*n*), 38 moved to abattoirs (*s*), 61 moved to saleyards (*s*), and 15 moved direct to farm (*s*)	Qualitative study among 12 commercial sheep producers in New South Wales (Australia)

9. Visitors to the property	Live vectors Fomites Vehicles	Proportion of pathways in which the FMD virus could spread from a sheep property including humans and dogs (Prop_Lvector_S), fomites (Prop_Fomite_S), and vehicles (Prop_Vehicle_S)	Live vectors: Pert (4, 20, 45) (*l*); fomites: Pert (1, 17, 43) (*f*); vehicles: Pert (4, 8, 25) (v) Prop_Lvector_S = *l*/(*l* + *f* + *v*) Prop_Fomite_S = *f*/(*l* + *f* + *v*) Prop_Vehicle_S = *v*/(*l* + *f* + *v*)	Qualitative study among 12 commercial sheep producers in New South Wales (Australia)

10. Disinfection of vehicles	Yes No	Probability of a contractor, feed truck, and stock movement vehicle being disinfected after coming into contact with sheep and sheep areas on the property (Prob_Disinfect_S)	Uniform (0.10, 0.33) 215 contractor vehicles, 22 clean their vehicles 12 producers, 4 producers ensure vehicles are clean	Qualitative study among 12 commercial sheep producers in New South Wales (Australia) (18)

11. Hygiene practices	Yes No	Probability that external personnel will take hygiene precautions between properties (Prob_Hyg_S)	Uniform (0.16, 0.53) 12 producers, 2 producers request clean equipment 870 producers, 465 ensure equipment is clean	Qualitative study among 12 commercial sheep producers in New South Wales (Australia) (9)

12. Neighboring enterprises	Cattle Sheep	Proportion of livestock enterprises surrounding sheep properties including cattle (Prop_Cattle_S) and sheep (Prop_Sheep_S) enterprises	Beta (*s* + 1, *n* − *s* + 1) 43 neighboring enterprises in total (*n*); 32 sheep enterprises (*s*); 11 cattle enterprises (*s*)	Qualitative study among 12 commercial sheep producers in New South Wales (Australia)

13. Shared boundary with neighboring stock	Yes No	Probability that a sheep flock will share a boundary with an adjacent livestock enterprise (Prob_nBound_S)	Beta (*s* + 1, *n* − *s* + 1) 50 boundaries (*n*); 34 boundaries adjacent with neighboring stock (*s*)	Qualitative study among 12 commercial sheep producers in New South Wales (Australia)

14. Exposure *via* aerosol	Yes No	Probability that FMD will spread to neighboring properties as a result of aerosol transmission from sheep to cattle (PI_S2C_S) and sheep to sheep (PI_S2S_S)	PI_S2C_S = Low (Uniform (0.05, 0.3)) PI_S2S_S = Very Low (Uniform (0.001, 0.05))	(17, 19)

15. Dead stock disposal method	Burnt/burial/lime No disposal	Probability that a carcass is disposed of by burial, incineration or lime (Prob_Dispose_S)	Beta (*s* + 1, *n* − *s* + 1) 12 producers (*n*), 8 producers burn, bury or lime dead stock (*s*)	Qualitative study among 12 commercial sheep producers in New South Wales (Australia)

16. Control of scavenging wildlife	Yes No	Probability that a producer will employ control strategies to prevent the scavenging of dead carcasses by wildlife (Prob_cScav_S)	Beta (*s* + 1, *n* − *s* + 1) 12 producers (*n*), 5 producers control scavengers (dogs and foxes) (*s*)	Qualitative study among 12 commercial sheep producers in New South Wales (Australia)

17. Wildlife found on property	Pigs Ungulates Kangaroos/foxes Vermin	Proportion of wildlife, which commercial NSW sheep producers are exposed to including pigs (Prop_wPig_S), ungulates (Prop_Ungulate_S), kangaroos and foxes (Prop_Native_S), and rodents (Prop_Vermin_S)	Beta (*s* + 1, *n* − *s* + 1) 30 potential contacts with wildlife (*n*): 3 reported contact with pigs (*s*), 3 reported contact with ungulates (*s*) 12 reported contact with kangaroos and foxes (*s*), and 12 reported contact with rodents (*s*)	Qualitative study among 12 commercial sheep producers in New South Wales (Australia)

18. Control of wildlife	Yes No	Probability that there is an appropriate control method for wildlife including pigs (Prob_cPig_S), ungulates (Prob_cUngulate_S), kangaroos and foxes (Prob_cNative_S), and rodents (Prob_cVermin_S)	Beta (*s* + 1, *n* − *s* + 1) Pigs: 3 reported contact (*n*), 1 reported control (*s*) Ungulates: 3 reported contact (*n*), 0 reported control (*s*); Kangaroos/Foxes: 12 reported contact (*n*), 5 reported control (*s*) Rodents: 12 reported contact (*n*), 5 reported control (*s*)	Qualitative study among 12 commercial sheep producers in New South Wales (Australia)

#### Sensitivity Analysis

Initially, a sensitivity analysis, using the @Risk Advanced Sensitivity Analysis (@RISK 6.0, Palisade Corporation, USA), was conducted to investigate the influence of some of the input parameters of the models to the overall probabilities of exposure and spread. Those input parameters representing husbandry, biosecurity, and animal health practices in which producers could actively improve upon were included in the sensitivity analysis. The specific aims of the sensitivity analysis were to identify which of these practices in each pathway of exposure and/or spread had the most influence in the probability of this pathway occurring and to investigate the potential impact of uncertainty around input parameters estimated using only the qualitative interviews. Once these influential and highly uncertain input parameters were identified, as a second step in the sensitivity analysis, several scenarios were used to further investigate the impact of the uncertainty around these parameters. For each input parameter identified, specific values of the probability distribution defining this parameter (median, minimum, and maximum) were used as fixed input values to run the corresponding model.

For the exposure assessment output, the influence of the following inputs was investigated: probability that a producer will introduce a FMD-infected sheep (Prob_Intro); appropriate quarantine of introduced sheep [Prob_Quar, defined as the combination of the probability of isolation (Prob_Isolate), the probability of isolated animal not sharing adjacent boundaries with susceptible animals (1-Prob_sBound), and the probability of a quarantine of ≥21 days (Prob_q21)]; probability of inspecting introduced sheep (Prob_Inspect); probability that clinical signs will be identified (Prob_ID); reporting to private veterinarian or government agency (Prob_Report); control of ungulates (Prob_cUngulate), kangaroos and foxes (Prob_cNative), and rodents (Prob_cVermin); probability that hygiene practices will be employed by visitors (Prob_Hyg); probability that vehicles will be disinfected (Prob_Disinfect); probability that sheep will graze with cattle (Prob_GwC); and probability that neighboring stock share a boundary (Prob_nBound).

For the consequence assessment output, the influence of the following input variables was investigated: probability that sheep graze with cattle (Prob_GwC_S); probability that FMD is detected in cattle (Prob_IDcattle_S); probability of early detection of clinical signs (Prob_EarlyID_S); probability that clinical signs will be identified (Prob_ID_S); probability of reporting to a private veterinarian or government agency (Prob_Report_S); probability of spread through sheep movements (Prob_Mov_S); proportion of sheep moved off a property to different destinations, including abattoirs (Prop_Abs_S), saleyards (Prop_Saleyard_S), and farms (Prop_Farm_S); probability that vehicles will be disinfected (Prob_Disinfect_S); probability that hygiene practices will be employed by visitors (Prob_Hyg_S); probability that neighboring stock share a boundary (Prob_nBound_S); probability of a carcass being disposed of by burial, incineration or lime (Prob_Dispose_S); probability that scavenging wildlife is controlled (Prob_cScav_S); and probability of control of ungulates (Prob_cUngulate_S), kangaroos and foxes (Prob_cNative_S), pigs (Prob_cPig_S), and rodents (Prob_cVermin_S).

The values for the input variables were allowed to vary from 0 to 1 in tenths, and each of these values was evaluated separately in a simulation of 5,000 iterations, keeping values for all other input variables fixed to their base value.

## Results

### Exposure Assessment

Assuming FMD is in the country and according to the pathways considered in this assessment, the median (5–95%) probability of exposure through an infected neighboring enterprise was estimated to be 0.710 (0.619–0.791). The main reasons for this high probability are the potential for aerosol exposure from neighboring infected pig properties as well as the direct contact of stock through boundary fences. This probability was similar to that estimated for exposure through the introduction of FMD-infected new stock (0.619; 0.541–0.698). This high probability is due to the transmission through direct contact before the infection is detected in introduced animals. The probabilities of exposure through wildlife and potential carriers (including visitors and vehicles) were estimated to be lower, with a median probability of 0.151 (0.085–0.239) and 0.235 (0.153–0.324), respectively.

### Probability of Spread

The probabilities of FMD spread from a commercial sheep flock in NSW through the five pathways considered in this assessment are shown in Table 6. According to the model, if FMD is introduced into a sheep property, the median probability of the virus not spreading to other properties or with limited spread due to early detection in cattle and or sheep was estimated to be 0.124 (0.066–0.173). Similar to the exposure assessment, the most likely spread pathway of FMD virus from an infected sheep property would be through neighboring enterprises mainly due to the high probability that sheep enterprises will have adjacent boundaries with other livestock properties, allowing for the transmission of the virus through direct contact or aerosol. Spread through wildlife was the next most likely pathway, with deer and goats being the most likely wildlife species spreading the FMD virus. Spread through the other pathways considered was less likely to occur.

**Table 6:** Predicted median (5 and 95%) of the probability of spread of foot-and-mouth disease from commercial sheep enterprises in New South Wales (Australia) through different pathways.

Spread pathways of FMD from commercial sheep flocks	Median^a^	5–95%
No spread/limited spread	0.124	0.066–0.173
Sheep movement	0.285	0.161–0.462
Carriers	0.315	0.171–0.527
Neighbors	0.603	0.504–0.698
Dead stock	0.168	0.070–0.312
Wildlife	0.523	0.404–0.638

*^a^Median probabilities of spread do not add up to 1 as all pathways can occur simultaneously*.

### Sensitivity Analysis

**Figure 6:**
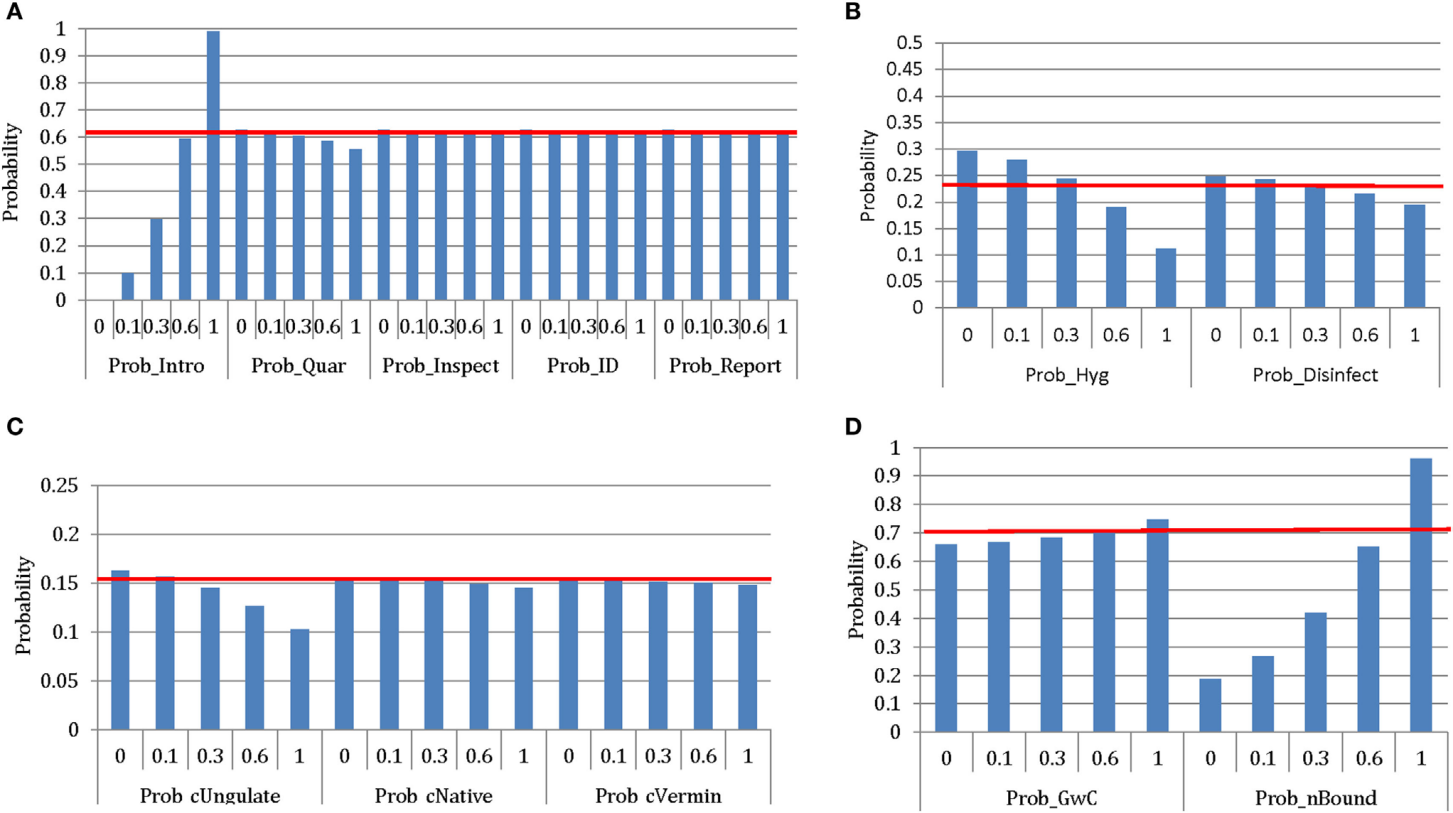
Results of the sensitivity analysis representing the influence of different input variables on the median (horizontal line) of exposure of a commercial sheep flock to foot-and-mouth disease (FMD) from **(A)** introduced stock; **(B)** potential carriers (humans, dogs, and vehicles); **(C)** wildlife; and **(D)** neighboring livestock enterprises in New South Wales, Australia. Results were obtained from a simulation of 5,000 iterations using @Risk’s Advanced Sensitivity Analysis. [Prob_Intro, probability that a producer will introduce a FMD-infected sheep; Prob_Quar, probability that new stock will be adequately quarantined (Prob_Isolate + Prob_sBound + Prob_q21); Prob_Inspect, probability that a producer will individually inspect new sheep; Prob_ID, probability that a producer will identify FMD-specific lesions as unusual; Prob_Report, probability that producer will contact veterinarian or government agency; Prob_Hyg, probability that external personnel will take hygiene precautions between properties; Prob_Disinfect, probability that vehicles will be disinfected before entering a property; Prob_cUngulate, probability that producers will control wild deer and goats; Prob_cNative, probability that producers will control kangaroos and foxes; Prob_cVermin, probability that producers will control rodents; Prob_GwC, probability that sheep graze with cattle; Prob_nBound, probability that a sheep flock shares a boundary with an adjacent livestock enterprise].

The initial sensitivity analysis investigated the influence of several input parameters on the probabilities of exposure and spread through different pathways. Figure 6 represents the sensitivity analysis for the exposure pathways. As expected, the most influential parameter for the probability of exposure through the introduction of infected new stock is the probability of introducing sheep (Prob_Intro). When the probability of introducing sheep into the property is decreased to 0.1 (base value, 0.63), there is a 6.3-fold decrease in the probability of exposure of susceptible sheep to FMD. Interestingly, other input values, such as the probability of appropriate quarantine (Prob_Quar), inspecting the newly introduced animals (Prob_Inspect), identifying clinical signs as unusual (Prob_ID), and reporting (Prob_Report), did not have as much influence as the probability of introducing new animals. In relation to the pathway of exposure through infected neighboring properties, the most influential parameter was the probability of the flock sharing a boundary with a neighboring property (Prob_nBound). When the probability of adjacent boundaries (allowing direct contact of stock) to neighboring stock is decreased to 0.1 (base value, 0.67), there is a 2.7-fold decrease in the probability of exposure to FMD through this pathway. Exposure through carriers (visitors, canines, and vehicles) is most influenced by the probability of visitors implementing appropriate biosecurity and hygiene practices (Prob_Hyg). When biosecurity and hygiene practices are implemented in full (base value, 0.35), there is a 2.1-fold decrease in the probability of exposure to FMD through this pathway. Given the presence of wildlife around properties would be very difficult to modify, the sensitivity analysis investigated the influence of appropriate control measures to minimize the contact of wildlife with domestic sheep. The most influential parameter was the probability of applying appropriate control measures for deer and goats (Prob_cUngulate); however, this influence was limited as shown in Figure 6, due to the potential for aerosol transmission.

Similar to the exposure sensitivity analysis, the most influential parameter for the spread of FMD to neighboring properties was the probability of having adjacent boundaries that allow for direct contact with neighboring stock and aerosol transmission of the virus. When this probability (Prob_nBound_S) is reduced to 0.1 (base value, 0.67), there is a 4.5-fold decrease in the probability of spread of FMD through this pathway. This spread probability is decreased by 11-fold when there are no adjacent boundaries allowing direct contact with neighboring stock, indicating the importance of appropriate segregation of properties. Spread of FMD through movement of sheep off the property is influenced by how frequently the producer is likely to move sheep off the property (Prob_Mov_S). A reduction in the probability of moving sheep will result in a lower probability of spread through this pathway, with a 3.5-fold reduction when the probability of sheep movement in a year is reduced to 0.1 (base value, 0.35).

Implementing appropriate measures to control wildlife would reduce the probability of spread through wildlife in a similar way to the exposure probability, as previously described. Spread through the distribution of infected carcass material by scavengers is highly influenced by the method of disposal of dead stock (Prob_Dispose_S). If carcass material is not appropriately disposed of, the probability of spread through scavengers would have a threefold increase, indicating the importance of appropriate disposal of dead stock for reducing the potential risk of disease spread. In this pathway, control measures implemented for scavenging wildlife (Prob_cScav_S) are also important. These measures need to be maintained in sheep properties as no control of scavengers will result in a twofold increase in the probability of spread through this pathway.

**Figure 7:**
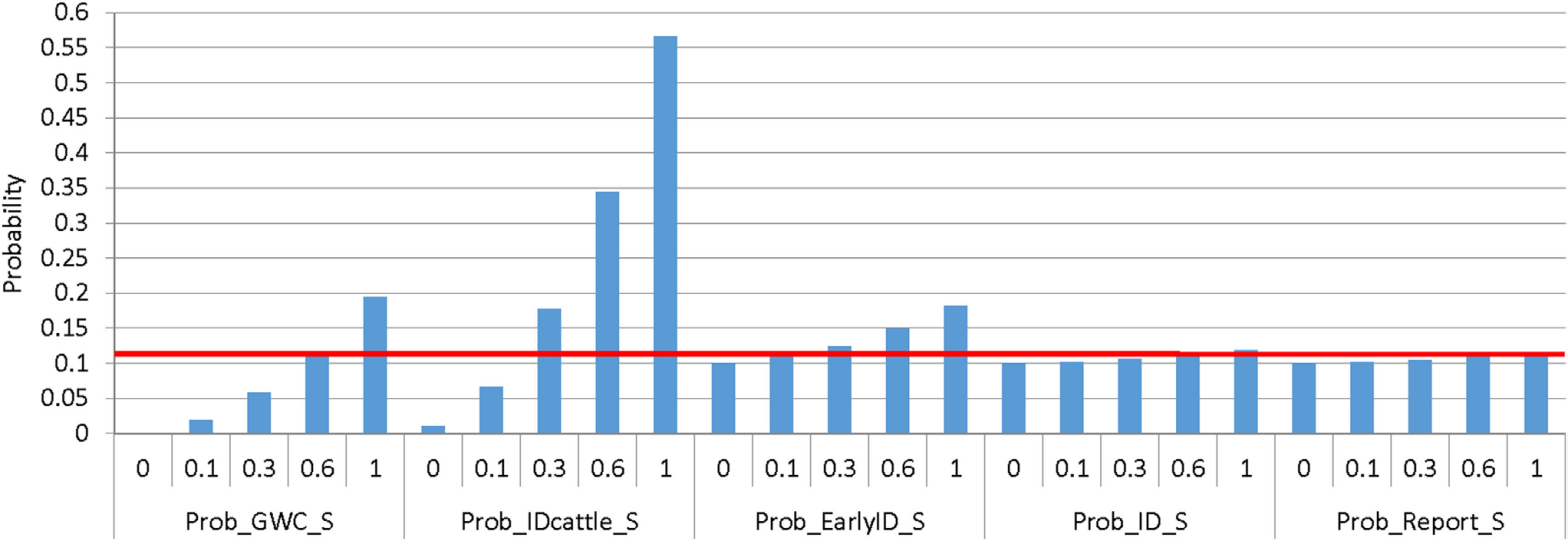
Results of the sensitivity analysis representing the influence of different input variables on the median (horizontal line) of foot-and-mouth disease (FMD) limited to no spread from a commercial sheep flock in New South Wales, Australia. Results were obtained from a simulation of 5,000 iterations using @Risk’s Advanced Sensitivity Analysis. (Prob_GwC_S, probability that sheep graze with cattle; Prob_IDcattle_S, probability that FMD will be detected in cattle; Prob_EarlyID_S, probability that FMD will be detected in sheep early enough to limit disease spread; Prob_ID_S, probability that a producer will identify FMD-specific lesions as unusual; Prob_Report_S, probability that producer will contact veterinarian or government agency).

As shown in Figure 7, the probability of detecting FMD in cattle (Prob_IDcattle_S) for sheep properties that also farm cattle is the most influential parameter to limit spread of FMD to other properties. If all producers are able to detect FMD lesions in cattle (base value 0.12), the probability of spread through all pathways would be reduced by half. Early identification of clinical signs in sheep would also decrease the probability of spread through all pathways; however, the impact of this input parameter is lower than the impact of detection in cattle.

**Figure 8:**
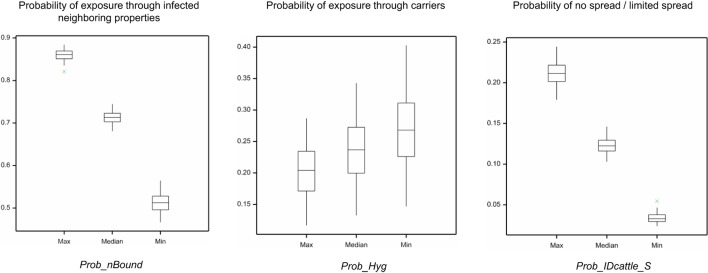
Results of the sensitivity analysis investigating the impact of the highly influential and uncertain input parameters to the outputs of the corresponding models (described in the title of each boxplot). Results were obtained from a simulation of 5,000 iterations run for each of the values used (minimum, median, and maximum) of the probability distribution of the input parameters. (Prob_nBound, probability that a sheep flock shares a boundary with an adjacent livestock enterprise; Prob_Hyg, probability that external personnel will take hygiene precautions between properties; Prob_IDcattle_S, probability that FMD will be detected in cattle).

From the initial sensitivity analysis, three input parameters that had been estimated mainly using the qualitative interviews with sheep producers were identified as highly influential on the model outcomes. These parameters were the probability of the flock sharing a boundary with a neighboring property (Prob_nBound; exposure through infected neighboring properties), the probability of visitors implementing appropriate biosecurity and hygiene practices [Prob_Hyg; exposure through carriers (visitors, canines, and vehicles)], and the probability of detecting FMD in cattle for sheep properties also farming cattle (Prob_IDcattle_S; no spread or limited spread). Each of the three corresponding models was run using the median, minimum, and maximum values of the probability distribution defining the input parameter and results are shown in Figure 8. As seen in the boxplots presented in this figure, the Prob_nBound and the Prob_IDcattle_S are the input parameters where the uncertainty around the estimates used have the most significant impact on the model outcomes. The median probability of exposure through infected neighboring properties would be 0.51, 0.71, and 0.86 when the minimum (0.42), median (0.68), and maximum (0.87) values of the Prob_nBound probability distribution are used. Similarly, the median probability of limited or no spread of FMD from a sheep property would be 0.03, 0.12, and 0.21 when the minimum (0.17), median (0.35), and maximum (0.53) values of the Prob_IDcattle_S probability distribution are used. In contrast, the Prob_Hyg has a more limited impact on the probability of exposure through carriers, with the median ranging from 0.26 to 0.20, when the minimum (0.04) and maximum (0.36) values of the distribution are used.

## Discussion

The aim of this study is to investigate the relative risk of disease introduction and spread among commercial sheep producers in NSW through different pathways using FMD as a case study and assuming that the virus is already in the country. Specifically, this study identified pathways of virus introduction into a sheep property and subsequent virus spread from this property and those biosecurity practices influencing these pathways. The outcome of each model is the overall probability of each pathway ending in exposure or spread and is interpreted as the probability of the pathway occurring due to exposure or spread happening in one or more of the limb events of the scenario tree representing this pathway. For example, if we consider the probability of exposure through wildlife, this exposure can occur due to feral pigs, ungulates, kangaroos/foxes, and/or vermin, with all of them considered independent to each other and being able to occur simultaneously. Given the models assumed independence of limb events in each scenario, the overall pathway probability could be overestimating the probability of exposure or spread. However, the independence assumption was applied in all pathway scenarios, and as such, the impact of this assumption on the ability to investigate the relative risk posed by different pathways is considered to be minimal. This study did not estimate the overall probability of exposure and spread for a sheep property, and as such, the proportional significance of each pathway was not considered. It is likely that in the event of an FMD outbreak, exposure and spread could occur through more than one pathway simultaneously. The study used data from a qualitative study with a cohort of 12 sheep commercial producers, which gathered information to support the development of the models, describing the pathways of exposure and spread of FMD and for estimating the input parameters to populate these models. Data from previous studies among commercial and smallholder sheep producers were also considered when populating the models to increase the validity and representativeness of the estimates (9, 11).

The exposure assessment component of this study identified four main pathways by which a commercial sheep flock could be exposed to FMD and estimated the probability of each of these pathways occurring. The model identified that transmission of the virus from an infected neighboring livestock enterprise would be likely to occur given the low biosecurity practices applied between properties sharing boundaries. This assumed that direct contact through the fence would result in instantaneous exposure to FMD, similar to previous studies modeling FMD transmission (20), in which farm-to-farm transmission was assumed to be instantaneous. However, this could be overestimating transmission between adjacent properties, especially in large land size properties where animals might not always go close to the boundary. Maller et al. (21) reported that peri-urban landholders in Australia identify neighbors failing to manage disease as the greatest risk of exotic disease outbreaks. Despite the importance of this pathway for disease spread, transmission of FMD through contact with neighboring enterprises is poorly described in the available literature.

Conversely, the role of sheep movements has been thoroughly described in the literature as a main contributor to the transmission of FMD between properties (5–7). Furthermore, sheep movements were identified as the main contributor to FMD transmission during the 2001 FMD outbreak in the UK (1). The current model identified the introduction of FMD-infected sheep as a likely source of exposure to FMD. Movement of animals to non-slaughter destinations, such as saleyards and other farms, have been identified as critical points for FMD transmission, as infected animals may be moved or sold before clinical disease becomes evident (7). This illustrates the importance of individual stock inspection, appropriate isolation, and the request of a SHS to minimize the risk of disease introduction. This study suggests that these practices could be improved among sheep producers.

This investigation also revealed that exposure of sheep to FMD through wildlife interactions and potential carriers were less likely scenarios. Feral pigs infected with FMD have been identified by Barclay (6) and Morgan (7) as influential to FMD transmission throughout Australia; however, only 25% of producers interviewed in the qualitative study had identified pig–sheep interactions. Geographical location of these producers might be influencing this proportion, and additional data from producers elsewhere in Australia are required to accurately estimate the potential risk posed by this pathway. Furthermore, available literature identified the influence of wild deer, Australian natives, foxes, and rodents on FMD transmission as low (5, 7). Similarly, the introduction of FMD into a sheep property through humans, equipment, and vehicles carrying the virus has also been identified as a low risk pathway in past outbreaks (1, 5–7, 16, 20).

The spread assessment considered six main scenarios in the spread of FMD from an infected sheep property to other susceptible livestock enterprises. Similar to the findings of the exposure assessment, spread to neighboring enterprises was identified as the most likely pathway of FMD spread. The qualitative interviews indicated that at least 68% of boundaries on sheep properties are adjacent to livestock on neighboring properties and that a quarter of these enterprises raise cattle. Spread of FMD between sheep and cattle is most likely to occur at close proximity as airborne transmission is less likely than that of pigs (1, 22). In addition, as cattle have been shown to have 12 times the infection risk of sheep, it is recommended that producers do not graze sheep and cattle together and that boundaries to neighboring livestock be kept free of susceptible animals (5, 6).

Wildlife was also found likely to influence the spread of FMD from an infected sheep property. Evidence in the literature on the effect of wildlife during transmission of FMD is limited. Schembri et al. (23) identified exposure to wildlife and feral pigs to domestic pigs as a major factor that may lead to the spread of an EAD, with approximately 35% of pig producers participating in this study reporting feral pigs, foxes, or bats seen on the property. Furthermore, Morgan (7) explained that feral pigs may facilitate the spread of FMD in Western NSW at a rate of approximately 2 km/d, and Animal Health Australia (24) recommends that inspection of stock be increased during periods of increased wildlife activity due to the potential for disease transmission. While wildlife may be responsible for the transmission of many endemic diseases in Australia, the qualitative interviews with sheep producers revealed that these producers were unlikely to associate wildlife with disease introduction or spread. This could be explained by a lack of knowledge of the potential risks posed by wildlife or the low presence of wildlife (feral pigs in particular) on these properties as results indicate.

Considering all input parameters within the pathway of exposure through the introduction of new stock, the sensitivity analysis revealed that the probability of introducing a sheep with FMD was the most influential parameter on the probability of exposure *via* this pathway. This indicates that introduction is more influential than quarantine and inspection, given the characteristics of FMD transmission, and the fact that exposure of susceptible species is likely to occur regardless of quarantine. Australian livestock producers have failed to identify introduction of diseased animals as a major risk to disease introduction in previous studies (6). Furthermore, over 20% of Australian sheep producers have been found to purchase stock from high-risk areas, such as saleyards (9). This further justifies the use of surveillance measures and inspection of salable stock at the site of purchase to reduce the risk of disease exposure to sheep on a naive property.

Sensitivity analysis also revealed that the probability of sharing adjacent boundaries between neighboring enterprises was the most influential parameter for both, exposure and spread scenarios through this pathway. The attitudes of Australian producers concur with this finding, as they believe that one of the greatest risks of an exotic disease outbreak arises from a neighbor’s failure to handle disease in their livestock (9). However, Barclay (6) found that only 14% of Australian producers maintain their boundary fences. This may arise from the perception that they have little control over the integrity of their neighbor’s boundary fences, as is the case for UK cattle producers (25). The probability of a sheep producer sharing adjacent boundaries with other livestock producers was estimated from the qualitative interviews, and given the restricted geographical area that these producers represent, uncertainty around this estimate is significant as reported in the sensitivity analysis, supporting the need for further investigating this practice among a more representative cohort of sheep producers. Nevertheless, this study emphasizes the importance of maintaining adequate boundary fences for the prevention of disease introduction and spread and the use double fencing or tree lanes to provide the best protection.

Besides the probability of boundary contact with neighboring livestock, sensitivity analysis also supports the use of appropriate biosecurity and hygiene practices of carriers, although relatively the importance of this pathway on the virus introduction is less significant than for other pathways investigated according to this model. Although the probability of applying appropriate biosecurity and hygiene practices among sheep producers was estimated from the qualitative interviews only, these findings are in agreement with Morgan (7) who identified hygiene practices as having minor influence to FMD exposure. However, having appropriate biosecurity and hygiene practices on farm is an easy measure to implement which would minimize the transmission of many sheep diseases. Taylor et al. (9) and the qualitative interviews indicate that personal hygiene for visitors when handling animals is of low priority to sheep producers (16.6 and 0.05%, respectively), and these results are similar to those among commercial beef producers, with approximately 14% of producers applying personal hygiene practices to prevent disease spread. Interestingly, this percentage was higher (approximately 40%) among smallholder livestock producers (10, 11). These findings suggest that these practices can be improved among livestock producers, and therefore, it is recommended that personal clothing and equipment are properly disinfected between properties to limit the transmission of disease.

While spread of FMD through carcass material was found to be least likely, correct disposal of dead stock was found to have a significant influence on the spread of disease through this pathway. One of the implications of incorrect disposal of carcasses is that foxes and dogs with access to these carcasses may facilitate the spread of infected material to neighboring properties. While Morgan (7) explained that the spread of carrion between farms is likely to be small, adequate disposal of carcasses will not only reduce the likelihood of spread through this pathway but may also decrease the overall accumulation of wildlife on a property. While disposal of carcasses is dependent on many environmental factors, burial and incineration are two methods that are most likely to reduce the risk of scavenging (26).

The majority of the input parameters used in this assessment were based on the data obtained by the qualitative interviews with sheep producers in NSW and Taylor et al. (9), with other literature used when required. However, information regarding the specific biosecurity practices of commercial sheep producers was limited, and most information was sourced from data gathered from the 12 producers interviewed. These interviews, although providing good in-depth information on biosecurity, might not be representing all commercial sheep producers in NSW, due to the restricted geographical area of the study and the selection methods used. The biosecurity practices of interviewed producers, who were considered proactive in regards to biosecurity, may have been above average. However, attitudes toward biosecurity and implementation of biosecurity practices among interviewed producers were diverse and were similar to those reported by of Palmer (8) and Taylor et al. (9), supporting the validity of the estimates. Nevertheless, results should be interpreted with caution, and further research to better understand biosecurity implementation among sheep producers in Australia is warranted. The significant influence of input parameters estimated using only the qualitative interviews, especially the probability of the flock sharing a boundary with a neighboring property and the probability of detecting FMD in cattle for sheep properties also farming cattle, supports the need for further research in this area to validate model outputs.

Aerosol transmission between properties was included in this assessment; however, as the focus of this study was to identify the relative importance of biosecurity practices of sheep producers, the influence of aerosol transmission on FMD exposure and spread was not explored. Furthermore, there is discord in the literature regarding the influence of aerosol transmission of FMD in Australia, and the extent of transmission can be affected by antigenic variation, climatic conditions, and topography (5–7, 27). Morgan (7) described a scenario in which FMD, present in a large group of asymptomatic sheep, could pose an increased risk of windborne transmission. The influence of stocking densities and feedlots on FMD transmission were not assessed in the current model, which may have resulted in an underestimation of exposure and spread. In addition, some other uncertainties influenced the outputs of this model. For example, there is no available information on the success of control strategies for wild ungulates, natives, and foxes; the role of deer and other wild ungulates in the transmission of FMD in previous outbreaks, such as the 2001 UK outbreak, is poorly understood (28); it is unknown whether pigs, natives, and foxes would travel between properties frequently enough to facilitate the transmission of FMD. As a result, the majority of wildlife contact data used in the model was sourced from the qualitative interviews with 12 producers, and therefore, the relevance of these results for Australian sheep production should be considered.

This study provides a model framework to investigate the significance of biosecurity practices for the risk of disease introduction into and spread from commercial sheep properties and to quantify the relative risk posed by different pathways of introduction and spread. Results from this FMD case study among commercial sheep producers in NSW suggest that potential for exposure to disease is most likely through neighboring enterprises and animal introductions. Findings indicate that implementation of biosecurity practices and animal health management among commercial sheep producers could be improved to minimize the risk of disease introduction and spread. Specific practices that producers should focus on are maintenance of boundary fences; inspection and isolation of introduced stock, preferably before movement onto the property; personal hygiene and equipment disinfection; and adequate disposal of dead stock. Further research on biosecurity practices among sheep producers in Australia would provide additional information to validate the findings of this study. In addition, further education of producers on the benefits of on-farm biosecurity and surveillance using effective communication strategies could minimize the risk of disease outbreaks in the future.

## Ethics Statement

Some of the data used to estimate the input parameters of the risk assessment model used in this study were drawn from qualitative interviews with sheep producers. The questionnaire and interview process were approved by the Human Ethics Low Risk Committee of Charles Sturt University, Australia (Approval 400/2015/28).

## Author Contributions

All authors contributed to the overall design of the study. MH-J and JF designed the semi-structured interviews used in the qualitative study to gather data from sheep producers, and RW and LR provided input to the interview tool. JF conducted, transcribed, and analyzed all interviews. RW supported JF with the analysis of the interviews. MH-J and JF developed the scenario trees and exposure and spread assessment simulation models and run all the models. MH-J and JF estimated the input values to be used in the models. JF and MH-J led the preparation of the manuscript, and RW and LR provided input to the manuscript.

## Conflict of Interest Statement

The authors declare that the research was conducted in the absence of any commercial or financial relationships that could be construed as a potential conflict of interest.
