# Supplementary material for: Assessing Biosecurity Risks for the Introduction and Spread of Diseases Among Commercial Sheep Properties in New South Wales, Australia, Using Foot-and-Mouth Disease as a Case Study
Source: Front Vet Sci. 2018 Apr 17;5:80. doi: 10.3389/fvets.2018.00080 (PMC5932351; doi:10.3389/fvets.2018.00080)

**Supplemental material**

1. **Exposure assessments**

A description of the nodes and input parameters for the four scenario trees used for the exposure assessment are provided below.

- 1. **Sheep introductions**

This exposure scenario tree describes the introduction of FMD into a sheep property via the introduction of an infected animal during the routine purchase of replacement stock.

- - 1. Introduces sheep

This node accounts for the likelihood that a producer will introduce an animal in a given year.

Past studies investigating the introduction practices of Australian sheep producers identify that 79% of flocks are self-replacing and that the only sheep introduced are ram purchases ([East and Foreman, 2011](#_ENREF_15)). This may decrease the risk of disease introduction; however with diseases such as FMD it may only require the introduction of a single infected animal to cause an outbreak on farm.

The qualitative data revealed that one out of 12 producers (8.3%) did not introduce any animals on an annual basis. Taylor et al. ([2011](#_ENREF_29)) conducted a study assessing the purchasing practices of NSW sheep producers and found that 216 out of 870 producers (24.8%) did not introduce any sheep in the past two years. As Taylor et al. ([2011](#_ENREF_29))’s data describes the introduction practices over two years, we believe the true proportion of producers not introducing animals in one year to be lower.

Therefore, data from Taylor et al. ([2011](#_ENREF_29))’s study and data from the qualitative interviews were considered together and used as the minimum value in a Pert distribution to estimate the probability of a sheep producer not introducing animals in the past 12 months (Prob_NoIntro). In contrast, according to a study by Hernández-Jover et al. ([2014a](#_ENREF_17)), up to 70% of sheep smallholders reported not introducing new animals in the previous 12 months. As our target producers are commercial sheep producers, this proportion is likely to be lower than that of smallholders. A maximum of 50% was used in the Pert distribution. As no data was available to accurately estimate the most likely point, the mid-point between the minimum and maximum was used. The probability of introducing sheep in the past 12 months (Prob_Intro) was estimated as 1 – Prob_NoIntro.

- - 1. Isolates new stock

This node accounts for the probability that a producer will isolate introduced stock when it is brought onto the property. Failure to isolate introduced animals will not only limit the producer’s ability to identify the disease, but will allow for the unmitigated transmission of disease via direct contact. The qualitative data revealed that of those that introduce new stock (n=11), eight producers (72.7%) isolate introduced animals. Taylor et al. ([2011](#_ENREF_29)) has also found that 575 out of 870 producers (66.1%) would quarantine new sheep on property for 7-10 days. This value does not account for those producers that quarantine for less than 7-10 days. Both of these values were combined and incorporated into a Beta distribution to account for uncertainty around this proportion.

- - 1. Shares boundary with main flock

This probability node accounts for the probability of an isolated animal being held in an enclosure adjacent to susceptible animals. According to Donaldson et al., 2001, the maximum distance the virus can transmit from a single infected sheep is 100 metres. Therefore, any susceptible species in enclosures adjacent to infected sheep will be exposed to the virus via aerosol or more likely, direct contact through enclosure fences. Conversely, assuming that a paddock is at least 100 metres in diameter, susceptible animals which do not share a fence boundary are unable to be exposed by direct contact and are less likely to be exposed via aerosol. The qualitative interviews revealed that of those producers that isolate new stock (n=8), six (75%) place stock in paddocks with a boundary adjacent to other sheep on the property. This data was incorporated into the model using a Beta distribution to account for uncertainty.

- - 1. Quarantine period of 21 days

This node accounts for the probability that a producer will quarantine introduced animals for a long enough period to allow for detection of FMD clinical signs. According to Morgan ([2006](#_ENREF_25)), sheep exposed to FMD may fail to develop clinical signs for a period of time. Animal Health Australia ([2014](#_ENREF_2)) has identified that the incubation period of FMD can range from 24 hours to 12 d, depending on the strain of the virus and the animal species involved. It is recommended in the literature that movement of animals be restricted for 21 d after contact with FMD as clinical signs are likely to develop over this period ([Morgan, 2006](#_ENREF_25)). The qualitative interviews have revealed that of those producers that quarantine new stock (n=8), 86% with isolate for a period of greater than 21 d. This was used as the maximum in a Pert distribution. Of the smallholders which introduce sheep into their property, 17% (n=13) reported isolating their sheep for a period greater than or equal to 21 d ([Hernández-Jover et al., 2014a](#_ENREF_17)). As our target producers are commercial sheep producers, this proportion was assumed to be higher than that of smallholders. Therefore, the proportion reported by smallholders was used as the minimum value in the Pert distribution. The mid-point between the minimum and maximum was used as the most likely value of the distribution.

- - 1. Clinical signs are apparent

This node accounts for the probability of FMD clinical signs developing in infected sheep. FMD can present with a wide spectrum of clinical signs in sheep, ranging from no clinical signs to highly noticeable disease ([Watson, 2004](#_ENREF_31)). The majority of available literature states that the disease is transient in sheep and that mild clinical signs may only be recognisable for 2.2 d ([Watson, 2004](#_ENREF_31); [Kitching et al., 2005](#_ENREF_20)). Furthermore, in studies investigating clinical signs in sheep, approximately 25% of sheep inoculated with FMD did not display clinical signs ([Watson, 2004](#_ENREF_31)). In other cases, the disease has presented as a high morbidity condition; however this morbidity is most often not observable unless sheep are congregated. This indicates that the probability of clinical signs being apparent in sheep would be Moderate. This qualitative estimate was transformed into a quantitative estimate using a Uniform distribution (Uniform(0.3,0.7)) following the semi-quantitative methodology for import risk analysis ([DAFF, 2004](#_ENREF_13)).

- - 1. Inspects new stock individually

This node assesses the probability that a sheep producer will individually inspect introduced sheep placed in quarantine. Many of the specific clinical signs associated with FMD cannot be adequately identified without close inspection of the individual sheep ([Watson, 2004](#_ENREF_31); [Animal Health Australia, 2014](#_ENREF_2)). Furthermore, many of the general clinical signs associated with lameness and ill-thrift may be associated with more common, less severe diseases such as endemic foot diseases ([Watson, 2004](#_ENREF_31); [Matthews, 2011](#_ENREF_24)). Therefore, producers must be willing to individually inspect introduced animals to identify lesions specific to FMD. Among producers interviewed, five (42.0%) reported individually inspecting stock if general clinical signs of disease are identified during routine husbandry procedures. However, individual inspection does not occur unless clinical signs are previously identified during group inspection. Taylor et al. ([2011](#_ENREF_29)) revealed that 780 out of 870 NSW sheep producers (89.7%) will inspect new stock on arrival; however, the method of inspection was not described in detail. The model estimated the probability of conducting individual inspections of new stock while in isolation based on this available data and a Uniform distribution was used in between these two proportions.

- - 1. Identifies clinical signs as unusual

This node accounts for the probability that a sheep producer will identify FMD-specific clinical signs as unusual signs. The most quoted clinical signs specific to FMD include blisters on top of the foot or between the claws, tongue and dental pad ([Animal Health Australia, 2014](#_ENREF_2)). The qualitative interviews revealed a wide range of knowledge amongst commercial sheep producers in relation to the clinical presentation of FMD. The majority of producers (n=6) identified that ‘blisters’ involving the tongue, mouth, feet or teats were indicative of an unusual or exotic disease. However, some producers did not know the clinical manifestations of FMD (n=4) and others indicated lesions in feet and mouth/lips without specifying the type of lesions. These data suggest that the probability of a sheep producer identifying clinical signs of FMD as unusual would be Moderate. This qualitative estimate was transformed into a quantitative estimate using a Uniform distribution (Uniform(0.3,0.7)) following the semi-quantitative methodology used for import risk analysis ([DAFF, 2004](#_ENREF_13)).

- - 1. Reports to private veterinarian or government agency

This node accounts for the probability that a sheep producer will contact a private veterinarian or government official once unusual clinical signs have been identified. This assumes that after this contact occurs the appropriate action will be taken to prevent further disease spread. The qualitative interviews revealed that nine out of 12 sheep producers (75%) would call a veterinarian as soon as unusual clinical signs are identified. Furthermore, Taylor et al. ([2011](#_ENREF_29)) found that 70% (n = 610) of producers would contact a veterinarian under similar circumstances. In addition, government officials were identified as secondary contacts by half of the producers. In contrast, eight producers also reported that they would contact a fellow producer and three producers indicated that would do nothing. In a study identifying the biosecurity practices of Australian beef commercial producers, most producers (98%) would contact a veterinarian in the event of unusual signs of disease and approximately 65% would contact a government veterinarian. However, almost 90% of producers also indicated that they would initially treat for disease themselves and over half of producers would contact another producer.

These data indicates that sheep producers are likely to call a private veterinarian or government official most of the time; however, it could be possible that some producers would choose to call someone else or do nothing, in which case spread of disease beyond the index farm would be more likely to occur. To account for this variability, data on veterinary contact from the qualitative interviews and Taylor et al. ([2011](#_ENREF_29)) was used as the most likely value of a Pert distribution. Given there where a number of producers who would contact another producer or do nothing, 0.5 was chosen as the minimum value of this distribution. The maximum reporting probability was set at 0.85.

- 1. **Wildlife**

This exposure scenario explores the exposure of a susceptible sheep to FMD via interactions with infected wildlife commonly found on NSW sheep properties.

- - 1. Wildlife on property

This category node allows for the differentiation of the type of wildlife in which commercial NSW sheep properties are exposed. The four main categories include feral pigs, ungulates such as deer and goats, native animals (kangaroos and foxes) and rodents. Other species present in NSW have been shown in the literature to have little to no role on potential transmission of FMD virus to sheep ([Kitching et al., 2005](#_ENREF_20); [Animal Health Australia, 2014](#_ENREF_2)). Producers in the qualitative interviews were asked what species of wildlife contacted their sheep or were present in sheep areas. All producers reported potential contact with wildlife species. Of these, three producers reported contact with feral pigs (Prop_wPigs), three producers reported contact with goats or deer (Prop_Ungulate), 12 identified both kangaroos and foxes contacting their sheep (Prop_Native) and 12 producers identified rodents on the property (Prop_Vermin). Considering all potential contacts, the corresponding proportion for each wildlife species was incorporated into the model using a Beta distribution.

- - 1. Wildlife control

This node accounts for the probability that there is an appropriate control method for wildlife considered in this assessment. If no adequate control measures are applied, direct or indirect contact of domestic animals with wildlife was assumed. This node did not apply to feral pigs as exposure to infected aerosol was assumed to occur independently of the control measures applied for this species. Infected pigs harbouring the virus will result in FMD exposure via aerosol spread, as pigs may excrete 1,000 to 3,000-times more virus particles in respiratory secretions than other susceptible species ([Barclay, 2005](#_ENREF_7); [Morgan, 2006](#_ENREF_25)). Qualitative interviews were used to identify how many producers had adequate control mechanisms to effectively decrease wildlife burden on the property, in order to minimise the potential exposure of FMD to susceptible sheep from infected wildlife. None of the producers that reported wild goat and deer around or on the property implement control strategies for these species (Prob_cUngulate). In regards to control of kangaroos and foxes, five out of 12 producers (42%) reported implementing multiple control strategies to limit the burden of these species on the property (Prob_cNative). FMD can also exist in the alimentary tract of rodents as a commensal. However, previous studies suggest that the role of rodents in the transmission of FMD virus to sheep is low when vermin control is implemented ([Morgan, 2006](#_ENREF_25); [Animal Health Australia, 2014](#_ENREF_2)). This node accounts for the probability that a producer will control the rodent population enough to limit FMD transmission. In the qualitative interviews, five out of 12 producers (42%) implemented rodent control on the property (Prop_cVermin). All of these probability values were incorporated into the model using Beta distributions to account for uncertainty.

- - 1. Supplementary feeding

This node accounts for the probability that supplementary feed will be provided to commercial sheep. The literature suggests that ingestion or inhalation of rodent faeces contaminated with FMD may be a viable source of FMD exposure ([Morgan, 2006](#_ENREF_25)). In properties with high rodent burden using supplementary feed, rodents are likely to be present in feed storage areas and consequently, feed could be contaminated with rodent faecal material. Half of the producers interviewed reported providing supplementary feed to their sheep on an annual basis. This data was incorporated into the model using a Beta distribution.

- - 1. Probability of infection

This node accounts for the probability of infection of FMD from a particular wildlife species to sheep after direct or indirect contact (exposure) has occurred. The probability of infection of sheep from exposure to FMD virus shed by feral pigs was considered to be High (PI_wPigs) as pigs secrete large amounts of virus in respiratory secretions ([Barclay, 2005](#_ENREF_7); [Morgan, 2006](#_ENREF_25)). Animals susceptible to FMD such as goats and deer may shed the virus in high quantities for a long period of time even after clinical signs have subsided; with goats being reported to carry the virus for up to 4 months ([Kitching et al., 2005](#_ENREF_20)). Therefore, the probability of exposure to the virus through deer and susceptible species is likely to be Moderate (PI_Ungulate). Natives and foxes are not thought to be susceptible to the virus and the literature is unclear as to whether the virus can be carried in the pharynx of these species ([Animal Health Australia, 2014](#_ENREF_2)). However, spread may also occur via fomite transfer (indirect transmission). The model assumes that the probability of virus transmission and subsequent infection of sheep from native animals is significantly lower than for wild goats and deer, and is estimated as Very low (PI_Native). Infection from rodents could occur after ingestion or inhalation of contaminated rodent faecal matter; however, rodents have not been reported to be implicated in the spread of FMD. Therefore, the probability of infection through rodents is estimated to be Very Low (PI_Vermin) ([Morgan, 2006](#_ENREF_25); [Animal Health Australia, 2014](#_ENREF_2)). These qualitative estimates were transformed to quantitative values using Uniform distributions following the semi-quantitative methodology for import risk analysis ([DAFF, 2004](#_ENREF_13)).

- 1. **Carriers**

This scenario explores the exposure of susceptible sheep to FMD through contact with live vectors (humans, dogs), fomites (equipment) and vehicles carrying the virus.

- - 1. Visitors to farm

This category node allows for the differentiation of potential pathways by which the FMD virus could be introduced into a sheep property through visitors and the quantification of their relative importance. Information on visitors, dogs and vehicles entering the sheep property among interviewed producers was used to estimate proportions required for this node. Live vectors, including humans and dogs, may harbour the virus in the nasopharynx for up to 24 hours after being exposed to an FMD infected animal ([Kitching et al., 2005](#_ENREF_20); [Morgan, 2006](#_ENREF_25); [Animal Health Australia, 2014](#_ENREF_2)). The qualitative interviews revealed that across 12 sheep properties, the number of humans (contractors used in the property) and dogs contacting a flock within the property ranged from four to 45, with a median of 20.

Fomites, which refer to equipment and clothing utilised by external personnel, is a recognised source of FMD virus ([Taylor et al., 2011](#_ENREF_29); [Animal Health Australia, 2014](#_ENREF_2)). Among interviewed producers, the range of the number of contractors used on the property was one to 43, with a median of 17. The qualitative interviews also allowed for the quantification of contractor, feed truck and stock movement vehicles entering a property on an annual basis. The range was from four to 25 vehicles per year with a median of eight. The range and median values were used in Pert distributions to estimate the number for each category and proportions were calculated and used in the model.

- - 1. Hygiene practices

Utensils and equipment have been found to play a major role in FMD transmission in sheep properties, emphasising the importance of maintaining appropriate biosecurity and hygiene precautions between properties ([Morgan, 2006](#_ENREF_25)). This node accounts for the probability that external personnel (contractors) will take these precautions and carry clean clothes and equipment. Taylor et al. ([2011](#_ENREF_29)) identified that approximately half of NSW sheep producers (465 out of 870) would ensure that any equipment brought onto the farm by external personnel is clean. In contrast, the qualitative interviews indicate that clean equipment and clothing was not routinely requested for contractors visiting sheep properties (two out of 12 producers). Given the difference between both studies and the lack of additional information, a Uniform distribution was used with both of these proportions.

- - 1. Disinfection

This node accounts for the probability of a contractor, feed truck or stock movement vehicle being disinfected before coming into contact with sheep and sheep areas on a property. A well-recognised method of FMD spread is through vehicles contaminated with lesion material, faeces, infected saliva or other secretions. Infection of subsequent stock will occur if the vehicle surfaces are not disinfected ([Morgan, 2006](#_ENREF_25)). Qualitative interviews reported that four out of 12 sheep producers (33%) would request vehicles to be disinfected before entering the property. There is no available data on vehicle disinfection practices of contractors in Australia. In England, Brennan and Christley ([2012](#_ENREF_11)) explored the disinfection practices of over 200 cattle contractors and found that only 10% would disinfect their vehicles routinely between properties. These values were incorporated into the model as the maximum and minimum values in a Uniform distribution respectively.

- - 1. Vehicle type

The probability of virus exposure and transmission from a contaminated vehicle to sheep in a property will depend on the type of vehicle, as different types of vehicles may be exposed to different amounts of infected material. This category node differentiates the most likely types of vehicles that may enter a sheep property. The qualitative interviews reported a total of 177 vehicles contacting the 12 sheep properties interviewed; 54 of these were livestock trucks (Prop_Courier), 107 were vehicles owned by external personnel and contractors (Prop_Contract) and 16 were feed trucks (Prop_Feed). This data was incorporated into the model using Beta distributions.

- - 1. Probability of infection

This node accounts for the probability of sheep being exposed and infected with FMD from fomites, live vectors (humans and dogs), courier vehicles, contractor vehicles and supplementary feed vehicles. According to literature, fomites and vehicles transporting infected animals are more likely to introduce FMD into a property than live vectors, such as humans and dogs ([Bourn, 2002](#_ENREF_9); [Morgan, 2006](#_ENREF_25); [Animal Health Australia, 2014](#_ENREF_2), [2015a](#_ENREF_3)). Although in some occasions fomites, such as equipment, have been found to play a minor role in FMD spread ([Morgan, 2006](#_ENREF_25)), several sources of literature identify infection via contaminated equipment ([Bourn, 2002](#_ENREF_9); [Morgan, 2006](#_ENREF_25); [Animal Health Australia, 2014](#_ENREF_2), [2015a](#_ENREF_3)). Humans and dogs may harbour the FMD virus in the nasopharynx for up to 24 hours post-exposure; however prolonged carriage in live vectors is considered unlikely ([Animal Health Australia, 2014](#_ENREF_2)). As previously mentioned, the amount of viral material on a vehicle is related to the extent of contact with infected animals shedding the virus. Sheep courier vehicles pose a higher risk of virus transmission than other vehicles ([Morgan, 2006](#_ENREF_25); [Brennan and Christley, 2012](#_ENREF_11)). Vehicles owned by contractors may be parked in sheep areas which can result in transfer of viral material from infected sheep onto the vehicle surface, but are less likely to transmit the virus than courier vehicles ([Brennan and Christley, 2012](#_ENREF_11)). Supplementary feed vehicles are the least likely to contact sheep flocks and so the probability of exposure is considered to be less than that of contractor vehicles. Qualitative estimates were used the estimate the probability of exposure and infection with FMD through these pathways. Fomites and courier vehicles were estimated to have a Moderate probability. Comparatively, live vectors and contractor vehicles were estimated to have a Low probability whilst supplementary feed vehicles were estimated as Very Low probability. These qualitative estimates were transformed to quantitative values using uniform distributions following the semi-quantitative methodology for import risk analysis ([DAFF, 2004](#_ENREF_13)).

- 1. **Neighbouring enterprises**

This scenario outlines the spread of FMD from an infected neighbouring property to susceptible animals in a sheep property.

- - 1. Neighbouring enterprises

This category node accounts for the different types of livestock enterprises surrounding sheep properties as the probability of exposure and infection of sheep might differ depending on the infected animal species. Information about neighbouring enterprises was sourced from the qualitative interviews. Producers were asked which enterprises were located in the areas surrounding their property. Of the 49 enterprises neighbouring the 12 sheep producers, six were piggeries in the area (Prop_nPig), 11 were cattle enterprises (Prop_Cattle) and 32 were sheep properties (Prop_Sheep). This data was incorporated into the model using Beta distributions.

- - 1. Flock graze with cattle

This probability node accounts for the probability that sheep flocks are held on the same pastures as cattle. Sheep are approximately 12 times less susceptible to being infected with FMD than cattle. Furthermore, cattle are at greater risk of infection via aerosol, due to a higher respiratory volume ([Barclay, 2005](#_ENREF_7); [Animal Health Australia, 2014](#_ENREF_2)). Therefore, the presence of cattle in a sheep property will increase the probability of infection of sheep as cattle are more likely to be infected via aerosol from neighbouring enterprises. This subsequently exposes and infects sheep grazing in the same area via direct contact. The qualitative data revealed that five out of 12 sheep producers (41.6%) graze cattle alongside with sheep. Furthermore, ([Hernández-Jover et al., 2014b](#_ENREF_18)) identified that approximately 60% (101 out of 175) beef commercial producers also have sheep on the property. Data from both studies was combined and incorporated into the model using a Beta distribution.

- - 1. Shared Boundary

In addition to aerosol transmission, FMD virus could be transmitted by direct contact from infected animals in neighbouring properties if both properties had a shared boundary. This node represents the probability that a sheep flock will share a boundary with an adjacent livestock enterprise (cattle or sheep). We assume that transmission of FMD by direct contact through the fence line will occur if susceptible sheep share a boundary with infected livestock. Qualitative interviews with 12 NSW sheep producers identified that 34 out of 50 property boundaries (68%) containing sheep have adjacent livestock on neighbouring properties, with these boundaries being separated by a single fence line. These values were incorporated into the model using Beta distributions.

- - 1. Aerosol exposure

This node accounts for the probability that sheep will be exposed to and infected with FMD virus via aerosol. Donaldson et al. ([2001](#_ENREF_14)) has published a table which compares the excretion and infection risk of pigs, cattle and sheep with respect to aerosolised FMD virus. Transmission from pigs to cattle is considered to pose the highest risk of transmission (High probability), followed by transmission from pigs to sheep (Moderate probability). Transmission from cattle to cattle and sheep to cattle is considered to be less likely (Low probability) and transmission from cattle to sheep and sheep to sheep poses the lowest risk (Very low probability). These qualitative estimates were transformed to quantitative values using uniform distributions following the semi-quantitative methodology for import risk analysis ([DAFF, 2004](#_ENREF_13)).

1. **Probability of spread**

The following nodes and input parameters were used to estimate the probability of spread of FMD virus to other sheep on the property and other properties once one sheep has been infected. This scenario involves the establishment of infection from a single infected animal in the flock and the most likely pathways in which the virus will spread from the infected premises. Five main spread pathways were considered, including: 1) Spread via live sheep movements; 2) Spread by visitors exiting the property; 3) Spread to neighboring enterprises; 4) Spread via infected carcass material; 5) Spread via various wildlife species; and, 6) No spread or limited spread. Figures representing each of the pathways of spread are provided below (Figure 1 to 5).

- 1. Flock graze with cattle

This node accounts for the probability that sheep flocks are held on the same pastures as cattle. Sheep are approximately 12-times less susceptible to FMD infection than cattle. Furthermore, cattle are at greater risk of infection via aerosol, due to a higher respiratory volume ([Barclay, 2005](#_ENREF_7); [Animal Health Australia, 2014](#_ENREF_2)). This is important as cattle might be more likely to be infected with the virus from the infected animal than sheep. In addition, clinical signs of FMD infection are more apparent in cattle than sheep ([Animal Health Australia, 2014](#_ENREF_2)), thus, disease could be more easily detected if cattle are infected in the property. The values for this node were calculated as for 1.4.2.

- 1. Detected in cattle

This node accounts for the probability that FMD will be detected in grazing cattle infected with FMD to limit the spread of the disease. Clinical signs are more severe and apparent in cattle than in sheep ([Animal Health Australia, 2014](#_ENREF_2)). Therefore, it is assumed that the virus will be detectable at an earlier time in cattle than in sheep. Hernández-Jover et al. ([2014b](#_ENREF_18)) determined that 27.6% (50 out of 181) of commercial beef producers inspect their cattle on a daily basis. However, as our primary target is commercial sheep producers who also farm cattle, daily inspection could be assumed to be less likely. The qualitative interviews revealed that among the 12 producers, five would never inspect their flock on a daily basis; six would inspect their stock daily for six weeks of the year; and, one producer would perform daily inspection for 18 weeks of the year. This suggests that for a significant period of the year animals are not inspected daily. The probability of a sheep producer conducting daily inspections in a week during a year was calculated combining the number of producers and the number of weeks per year these would conduct daily inspections. Beta distributions were used to incorporate these proportions. The probability of a sheep producer detecting FMD in cattle on their property was then calculated using a Uniform distribution with data from Hernández-Jover et al. ([Hernández-Jover et al., 2014b](#_ENREF_18)) as the maximum value and the qualitative interviews as the minimum value.

- 1. Clinical signs apparent in sheep

This node accounts for the probability that infected sheep will develop clinical signs of FMD. Variability in strain and climatic conditions can cause the clinical signs of FMD in sheep to present as no clinical signs to a highly noticeable disease ([Watson, 2004](#_ENREF_31); [Morgan, 2006](#_ENREF_25)). The values for this node were calculated as for 1.1.5.

- 1. Early detection of clinical signs

This node accounts for the probability that FMD will be detected in sheep early enough to limit the spread of disease. The early detection of clinical signs relies highly on the daily inspection practices of sheep producers ([Watson, 2004](#_ENREF_31); [Morgan, 2006](#_ENREF_25)). As explained in node 2.2., the qualitative interviews revealed that daily inspection varies during the year, with some producers conducting these inspections more often than others, depending on time of year and lambing status. The probability of a sheep producer detecting FMD clinical signs was estimated based on the probability of a sheep producer conducting daily inspections in a week during a year, as explained in node 2.2.

- 1. Identifies clinical signs as unusual

For reporting to occur, the producer should identify the FMD clinical signs as unusual. This node accounts for this probability. The most quoted clinical signs specific to FMD include blisters on top of the foot or between the claws, tongue and dental pad ([Animal Health Australia, 2014](#_ENREF_2)). The values for this node were calculated as for 1.1.7.

- 1. Reports to private veterinarian or government agency

This node accounts for the probability that a sheep producer will contact a private veterinarian or government official once unusual clinical signs have been identified. This assumes that after this contact occurs, the appropriate action will be taken to prevent further disease spread. The values for this node were calculated as for 1.1.8.

- 1. Spread of FMD

The spread model assumes that if FMD is not detected the virus would spread from the sheep flock through different pathways. This node represents these potential pathways and the probability that each spread pathway will occur. A review of the available literature on the spread of FMD has enabled the categorisation of most likely spread pathways, including sheep movements, neighbouring enterprises, visitors, wildlife and dead stock. Given all sheep producers interviewed reported having neighbouring properties with sheep and/or cattle, if FMD is not detected in the infected property, it is assumed that the virus could spread to these neighbouring properties, and the magnitude of the spread would depend on the exposed livestock species (see 2.12), the shared boundaries (see 2.13) and the aerosol spread (see 2.14). Similarly, all sheep producers reported having wildlife in their property, thus spread of the FMD virus through these pathways (wildlife and dead stock) could occur and would be dependent on the wildlife species, the carcass disposal methods and control measures for wildlife (see 2.15 to 2.18).

Direct contact between animals is the most likely means of virus transmission ([Kitching et al., 2005](#_ENREF_20)). Movement of infected animals has been identified by several sources of literature as the most influential factor in spread of FMD ([Barnett and Cox, 1999](#_ENREF_8); [Bourn, 2002](#_ENREF_9); [Morgan, 2006](#_ENREF_25); [Animal Health Australia, 2014](#_ENREF_2)). Furthermore, sheep movement in particular was identified as the main contributor to FMD spread during the 2001 FMD outbreak in the UK ([Bourn, 2002](#_ENREF_9)). The probability of spread through movement of sheep is dependent upon the frequency of movement of infected sheep to other destinations where susceptible sheep are present. The qualitative interviews were used to assess the frequency of sheep movements off the property on an annual basis. The interviews revealed that the highest proportion of producers move sheep off property five to 10 and 11 to 20 times a year (n = 4). Taylor et al. ([2011](#_ENREF_29)) also assessed the number of times producers had sold sheep other than to slaughter, revealing that the highest proportion of producers that sold sheep would sell 1 to 4 times every two years. Among the 12 producers interviewed, one producer reported moving sheep off property once per year, three between two and four times per year, four between five to 10 times per year, four between 11 to 20 times per year and one producer reported moving sheep off property more than 20 times per year. Each of these categories was assigned a qualitative risk value: extremely low, very low, low, moderate and high, respectively. These qualitative estimates were transformed into quantitative estimates using Uniform distributions following the semi-quantitative methodology for import risk analysis ([DAFF, 2004](#_ENREF_13)), and subsequently used to calculate a combined probability of spread through sheep movement (Prob_Mov_S), using the proportion of producers in each category.

Vehicles, equipment and personnel working with sheep (carriers) have also been identified by several sources of literature as a viable transmission pathway for FMD ([Kitching et al., 2005](#_ENREF_20); [Morgan, 2006](#_ENREF_25); [Animal Health Australia, 2014](#_ENREF_2)). The spread of FMD through potential carriers is dependent on the frequency of visitors entering the property. Contamination of vehicles, equipment or clothing surfaces with animal material carrying the virus in the absence of disinfection and hygiene practices can potentially lead to spread of FMD ([Morgan, 2006](#_ENREF_25); [Animal Health Australia, 2014](#_ENREF_2)). The virus can also survive in the nasopharynx of humans and animals for up to 24 hours and can be excreted in the vicinity of susceptible animals by sneezing, coughing or breathing ([Morgan, 2006](#_ENREF_25)). The qualitative interviews revealed that the most common frequency of movement of potential carriers was five to 10 times per year (n=5). The remainder of the 12 producers revealed that carriers enter the property two to four times a year (n=2), 11 to 20 times a year (n=2) and >20 times a year (n=2). These categories were assigned a qualitative risk value: low, very low, moderate and high respectively. These qualitative estimates were transformed into quantitative estimates using Uniform distributions following the semi-quantitative methodology for import risk analysis ([DAFF, 2004](#_ENREF_13)), and subsequently used to calculate a combined probability of spread through carriers (Prob_Visitor_S), using the proportion of producers in each category.

- 1. Destination

This category node differentiates the most likely destination of a consignment of sheep when moved off a property. One of the most likely factors in disease transmission is the movement of an infected animal through saleyards and non-slaughter destinations ([Morgan, 2006](#_ENREF_25)). Animals sent straight to an abattoir are the least likely pathway of spread of the virus via direct contact and may be considered an end-point in direct disease transmission. Movements involving non-slaughter, typically through saleyards and direct to farm, are most likely to cause transmission of FMD. Saleyards in particular, pose an additional risk of transmission due to the increased stocking density of animals and the multifocal destination of animals. Qualitative interviews revealed that of all sheep movements (n=144), 38 are movements to abattoirs (Prop_Abs_S), 61 movements are to saleyards (Prop_Saleyard_S) and 15 movements are direct to farm (Prop_Farm_S). These values were incorporated into the model using Beta distributions.

- 1. Visitors

This category node represents the different categories of visitors which may be associated with FMD spread from a commercial sheep property, including live vectors (humans and dogs), fomites and vehicles. This node assumes that all external personnel, equipment and vehicles will work with susceptible animals on other properties when leaving the infected property, and spread could subsequently occur. The values for this node were calculated as for 1.3.1.

- 1. Disinfection

This node accounts for the probability of a contractor, feed truck or stock movement vehicle being disinfected after coming into contact with FMD infected sheep. Transmission via vehicles is a well-recognised method of FMD spread, through the contamination of vehicles with lesion material, faeces, infected saliva or other secretions. Infection of subsequent stock will occur if the vehicle surfaces are not disinfected ([Morgan, 2006](#_ENREF_25)). The values for this node were calculated as for 1.3.3.

- 1. Hygiene practices

This node accounts for the probability that external personnel (contractors) will take hygiene precautions and use clean clothes and equipment after contacting infected sheep. The values for this node were calculated as for 1.3.2.

- 1. Neighbouring enterprise

This category node accounts for the different types of neighbouring enterprises surrounding an infected sheep property. The extent of FMD spread will vary depending on the species present on neighbouring properties. Neighbouring piggeries have not been included as the likelihood that pigs will be infected by aerosol from sheep is negligible. Furthermore, contemporary intensive piggeries are unlikely to encounter direct contact with sheep ([Donaldson et al., 2001](#_ENREF_14)). Cattle are more susceptible to aerosol spread than sheep, thus those neighbouring properties with cattle will be at higher risk of FMD transmission than those with sheep alone ([Donaldson et al., 2001](#_ENREF_14); [Barclay, 2005](#_ENREF_7)). Information regarding neighbouring enterprises was sourced from the qualitative interviews. Producers were asked which enterprises were located in the areas surrounding their property. Of the 43 livestock enterprises neighbouring the 12 sheep producers, 11 were cattle enterprises (Prop_Cattle_S) and 32 were sheep properties (Prop_Sheep_S). This data was incorporated into the model using Beta distributions.

- 1. Adjacent boundary with neighbouring enterprise

In addition to aerosol transmission, FMD virus could be transmitted by direct contact from infected animals in neighbouring properties if both properties had a shared boundary. This node represents the probability that a sheep flock will share a boundary with an adjacent livestock enterprise (cattle or sheep). We assume that transmission of FMD by direct contact through the fence line will occur if infected sheep share a boundary with susceptible livestock. These values were calculated as for 1.4.3.

- 1. Aerosol exposure

This node accounts for the probability that the disease will be spread to neighbouring properties as a result of aerosol transmission. Donaldson et al. ([2001](#_ENREF_14)) has published a table which compares the excretion and infection risk of pigs, cattle and sheep with respect to aerosolised FMD virus. Transmission from sheep to cattle is considered to be more likely (Low probability) than from sheep to sheep, which poses the lowest risk (Very low probability). These qualitative estimates were transformed to quantitative values using uniform distributions following the semi-quantitative methodology for import risk analysis ([DAFF, 2004](#_ENREF_13)).

- 1. Dead stock disposal method

This probability node differentiates the methods in which a carcass is disposed on a commercial NSW sheep property. Dead stock carrying the FMD virus may be responsible for transfer of the virus through viscera as these do not undergo a post-mortem pH change significant enough to neutralise the virus (in comparison to muscle tissue) ([Australian Quarantine and Inspection Service, 1999](#_ENREF_6)). The incineration or burial of infected carcasses will destroy viral material or prevent access to scavengers or susceptible stock, respectively. Covering carcass material in lime may also be sufficient to cause a pH change significant enough to neutralise FMD virus ([Animal Health Australia, 2015b](#_ENREF_4)). The disposal methods of the 12 producers interviewed revealed that eight of these producers will dispose of dead stock by burial, incineration or lime (67%) and that four of these producers may do nothing for carcass disposal (33%). These values were incorporated into the model using Beta distributions.

- 1. Scavenger control

This node accounts for the probability that a producer will employ control strategies adequate enough to prevent the scavenging of carcass material. Scavengers, including foxes and dogs, may facilitate spread of FMD by carrying carcass material onto other properties with susceptible animals ([Morgan, 2006](#_ENREF_25)). Therefore, adequate control of scavenging wildlife will minimise the risk of FMD spread via carrion. Qualitative interviews were used to identify the proportion of producers which implemented wildlife control strategies. Of 12 NSW commercial sheep producers, five reported implementing multiple control strategies to reduce scavenging species on the property. These values were incorporated into the model using Beta distributions.

- 1. Wildlife

This category node allows for the differentiation of the type of wildlife in which commercial NSW sheep properties are exposed. The four main categories include feral pigs, ungulates such as deer and goats, native animals (kangaroos and foxes) and rodents. Other species present in NSW have been shown in the literature to have little to no role on the potential transmission of FMD virus ([Kitching et al., 2005](#_ENREF_20); [Animal Health Australia, 2014](#_ENREF_2)). Producers in the qualitative interviews were asked what species of wildlife contacted their sheep or were present in sheep areas. All producers reported potential contact with wildlife species. Of these, three producers reported contact with feral pigs (Prop_wPig_S), three producers reported contact with goats or deer (Prop_Ungulate_S), 12 identified both kangaroos and foxes contacting their sheep (Prop_Native_S)) and 12 producers identified rodents in feed storage areas on the property (Prop_Vermin_S). The corresponding proportions were calculated as for 1.2.1.

- 1. Wildlife Control

This node accounts for the probability that there is an appropriate control method for wildlife considered in this assessment. The probabilities of having appropriate control methods for ungulates, native animals/foxes and vermin were estimated as for 1.2.2. In addition, the impact of control methods for feral pigs was also considered for the potential spread of the virus. Of the three interviewed producers who reported pigs on their property, one employs mechanisms for pig control. This value was incorporated into the model using a Beta distribution.


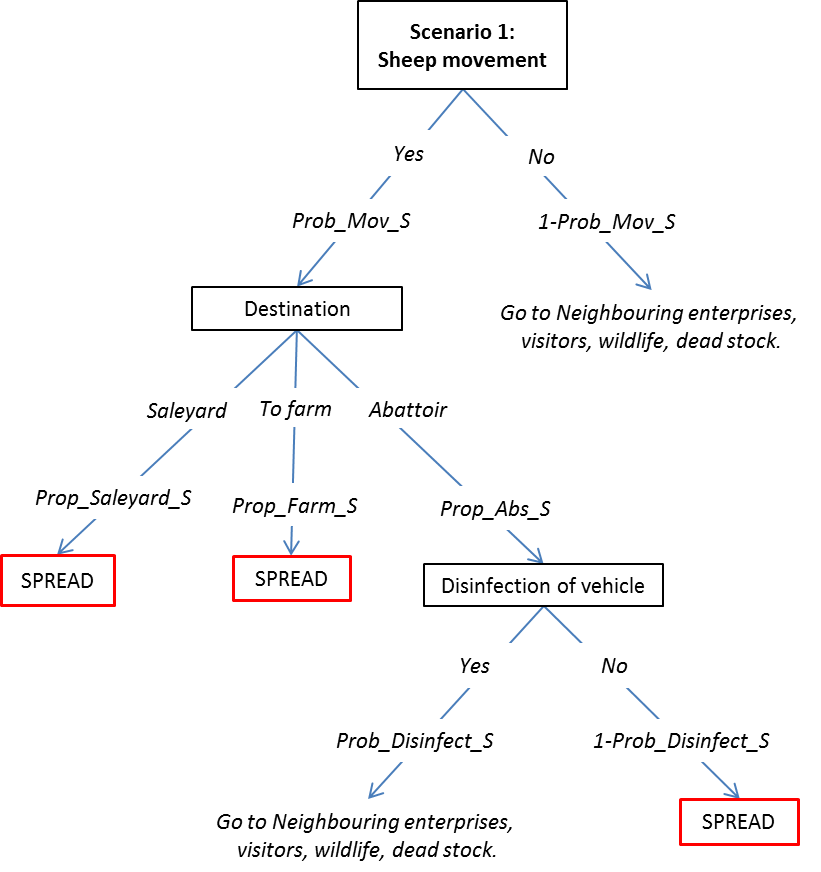
Figure 1. Scenario tree representing the spread of FMD from an infected sheep property to a susceptible livestock enterprise through movement of sheep; (Prob_Mov_S = Frequency of sheep movements off the property; Prop_Abs_S = Proportion of sheep moved to abattoirs; Prop_Saleyard_S = Proportion of sheep moved to saleyards; Prop_Farm_S = Proportion of sheep moved to farm; Prob_Disinfect_S = Probability that a stock movement vehicle will be disinfected after contacting FMD-infected sheep).


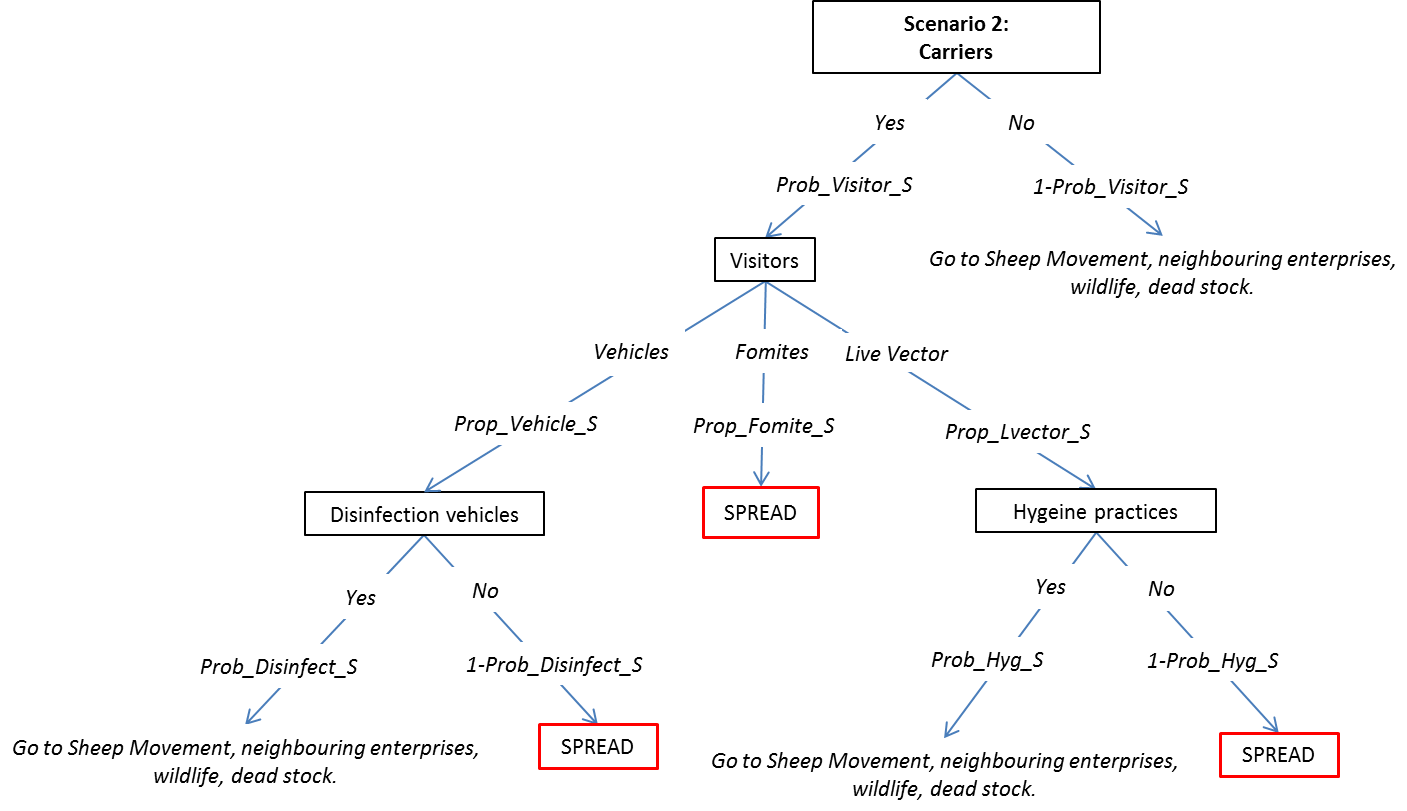


Figure 2. Scenario tree representing the spread of FMD from an infected sheep property to a susceptible livestock enterprise through carriers (humans, dogs and vehicles); (Prob_Visitor_S = Frequency of visitor movements on and off the property; Prop_Vehicle_S = Proportion of vehicles entering a property; Prop_Fomite_S = Proportion of fomites entering a property; Prop_Lvector_S = Proportion of humans and dogs entering the property; Prob_Disinfect_S = Probability that a vehicle will be disinfected after contacting the property; Prob_Hyg_S = Probability that personnel will take hygiene precautions between properties).


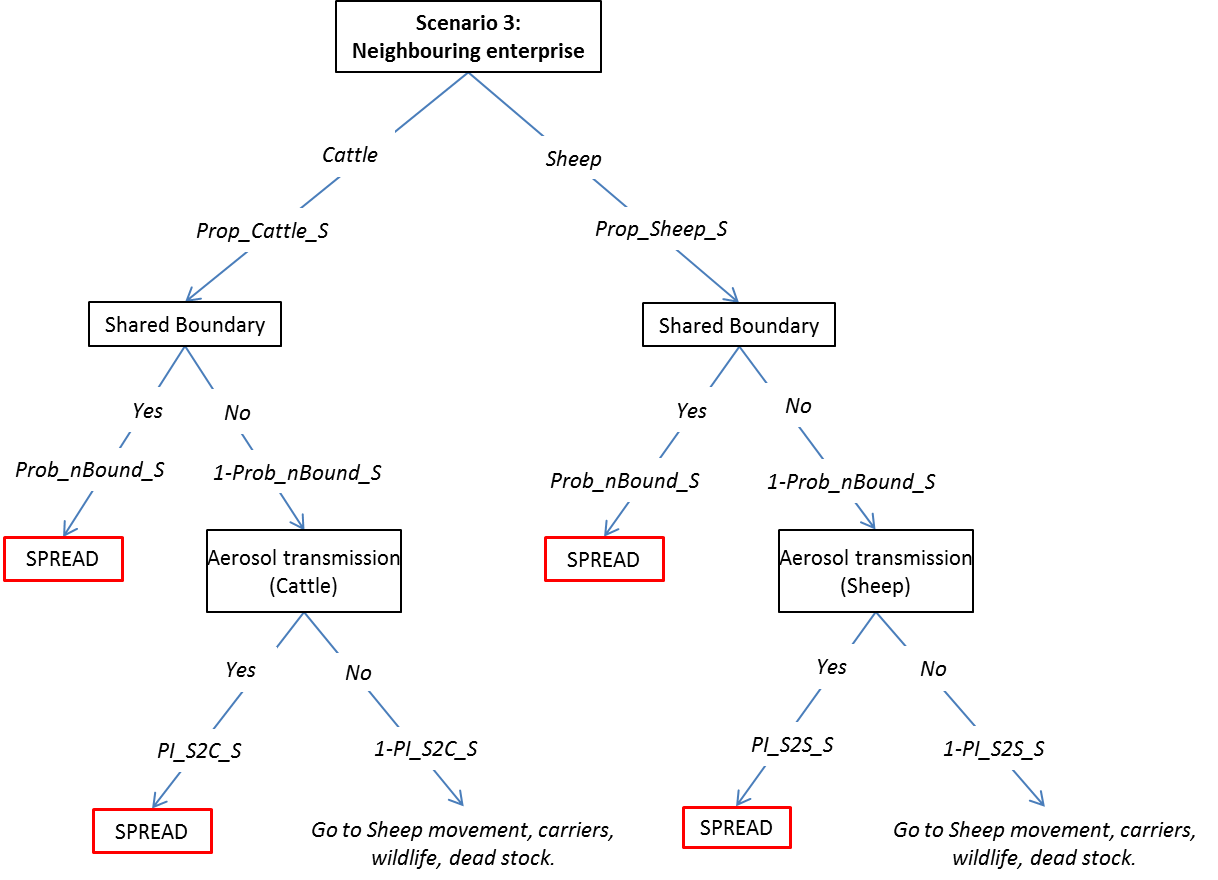


Figure 3. Scenario tree representing the spread of FMD from an infected sheep property to a neighbouring enterprise; (Prop_Cattle_S = Proportion of neighbours with cattle; Prop_Sheep_S = Proportion of neighbours with only sheep; Prob_nBound_S = Probability that a sheep flock shares a boundary with an adjacent livestock enterprise; PI_S2C_S = Probability of aerosol transmission from sheep to cattle; PI_S2S_S = Probability of aerosol transmission from sheep to sheep).


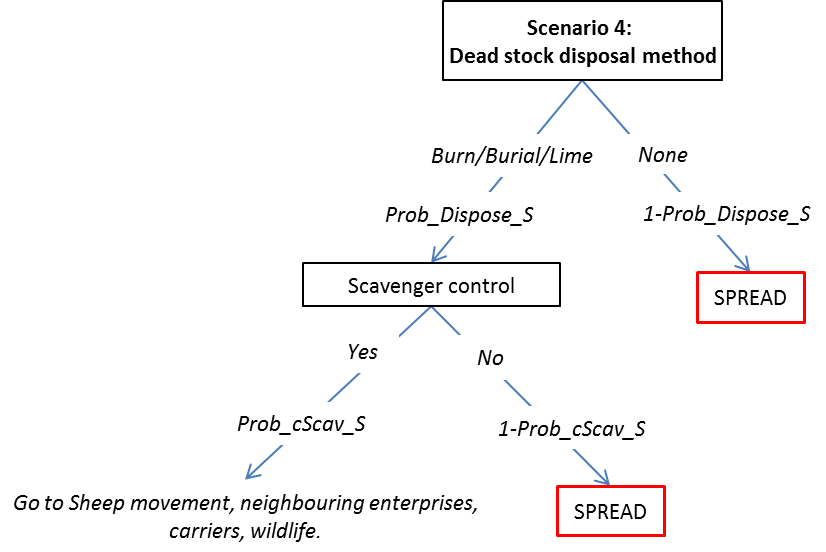


Figure 4. Scenario tree representing the spread of FMD from an infected sheep property to a susceptible livestock enterprise through carrion; (Prob_Dispose_S = Probability that a carcass is disposed of by burial, incineration or lime; Prob_cScav_S = Probability that a producer will employ control strategies to prevent the scavenging of dead carcasses by wildlife).

Figure 5. Scenario tree representing the spread of FMD from an infected property to a susceptible livestock enterprise through wildlife; (Prop_wPig_S = Proportion of wild pigs on the property; Prop_Ungulate_S = Proportion of deer and goats on the property; Prop_Native_S = Proportion of kangaroos and foxes on the property; Prop_Vermin_S = Proportion of rodents on the property; Prob_cUngulate_S = Probability that producers will control wild deer and goats; Prob_cNative_S = Probability that producers will control kangaroos and foxes; Prob_cPig_S = Probability that producers will control wild pigs; Prob_cVermin_S = Probability that producers will control rodent).
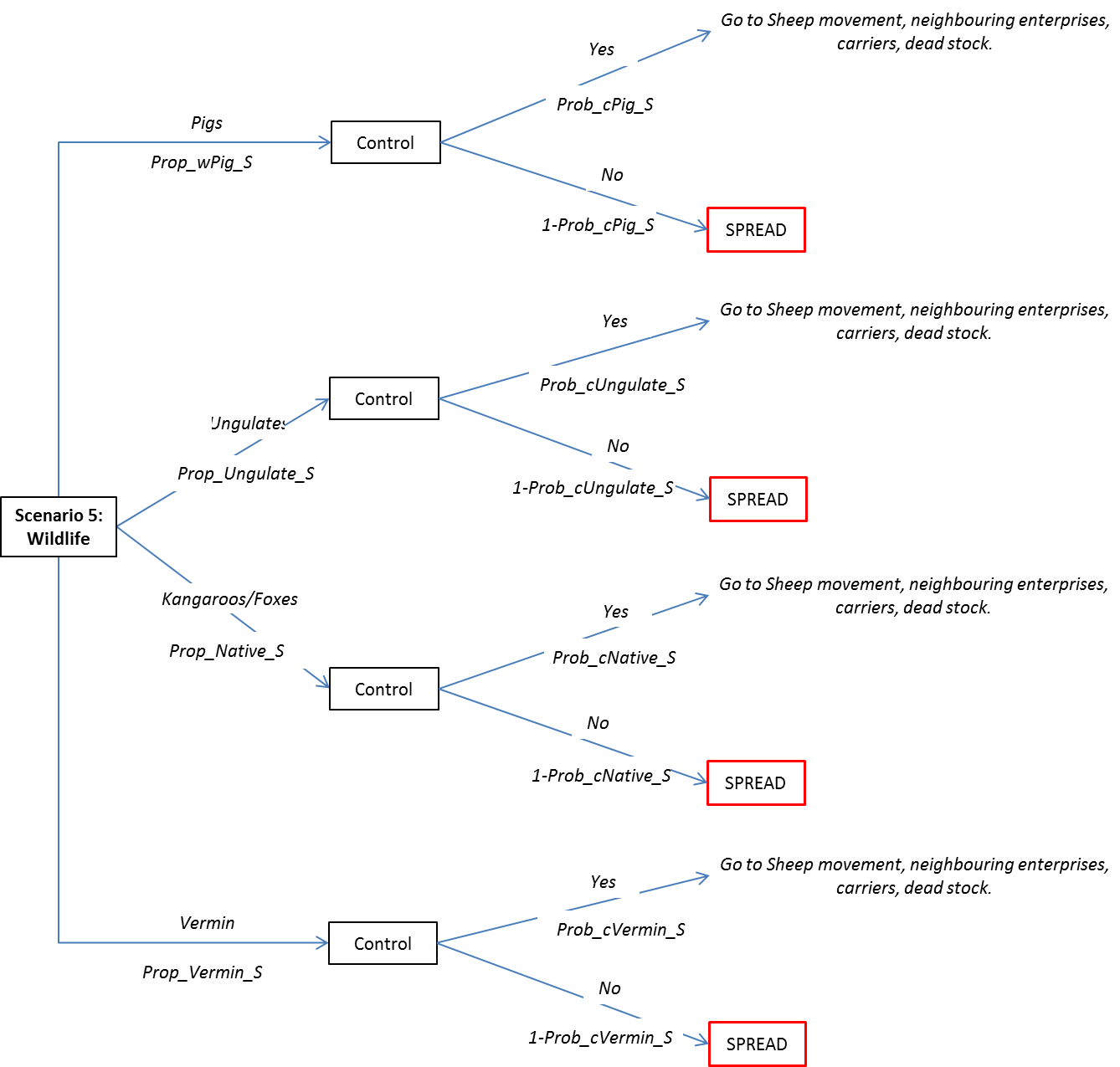

Supplement: Supplementary file 1 [file data_sheet_1.docx]
